# Expression profiling in a mammalian host reveals the strong induction of genes encoding LysM domain-containing proteins in *Enterococcus faecium*

**DOI:** 10.1038/s41598-018-30882-z

**Published:** 2018-08-17

**Authors:** Margherita Cacaci, Caroline Giraud, Loic Leger, Riccardo Torelli, Cecilia Martini, Brunella Posteraro, Valentina Palmieri, Maurizio Sanguinetti, Francesca Bugli, Axel Hartke

**Affiliations:** 1 Normandie Univ, UNICAEN, U2RM-Stress and Virulence, 14000 Caen, France; 2 Institute of Microbiology, Università Cattolica del Sacro Cuore, Fondazione Policlinico Universitario IRCCS Agostino Gemelli, 00168 Rome, Italy; 3 Institute of Public Health (Section of Hygiene), Università Cattolica del Sacro Cuore, Fondazione Policlinico Universitario IRCCS Agostino Gemelli, 00168 Rome, Italy; 4 Physics Institute, Università Cattolica del Sacro Cuore, Fondazione Policlinico Universitario IRCCS Agostino Gemelli, 00168 Rome, Italy

## Abstract

*Enterococcus faecium* is an important health care-associated pathogen that is difficult to treat due to the high level of antibiotic resistance of clinical isolates. The identification of new potential therapeutic targets or vaccination strategies is therefore urgently needed. In this regard, we carried out a transcriptomic analysis of the *E. faecium* vancomycin-resistant strain AUS0004, comparing the gene expression of bacteria grown under laboratory conditions and bacteria isolated from an infection site. This analysis highlighted more than 360 genes potentially induced under infection conditions. Owing to their expression profiles, four LysM domain-containing proteins were characterized in more detail. The EFAU004_01059, 1150 and 494 proteins are highly homologous, whereas EFAU004_01209 has a unique domain-architecture and sequence. The analysis of corresponding mutants showed that all LysM proteins played relevant roles in the infection process of *E. faecium* in mice. The EFAU004_01209 mutant also displayed profound morphological modifications, suggesting it has a role in cell wall synthesis or cell division. Furthermore, the adhesion to kidney cells and growth of the mutant was affected in human urine. All these phenotypes and the surface exposure of EFAU004_01209 identify this protein as an interesting new drug target in *E. faecium*.

## Introduction

Enterococci are Gram-positive bacteria that colonize several ecological niches, including the guts of mammals and numerous other animals^1^. These bacteria have emerged as important nosocomial pathogens due to their multiple antibiotic resistances^2^. *Enterococcus faecalis* and *Enterococcus faecium* are the third and fourth most commonly isolated nosocomial pathogens worldwide, causing up to 14% and 16% of hospital acquired infections in the US and Europe respectively^3–5^. *E. faecium* infections, in particular, have become a major concern, since their resistance to vancomycin and ampicillin has increased up to almost 100% in some clinical institutions in the US, and a similar rise in resistances has recently been observed in Europe^6–8^. The ability of *E. faecium* to survive under a range of adverse environmental conditions and the dramatic increase in the antibiotic resistance of clinical isolates highlight the need for the development of alternative treatments and prevention strategies^9,10^. Well expressed and abundant proteins, located on the surface of bacteria during the infection process, could be potential targets for new drugs or vaccines^11–13^. To identify these surface proteins in *E. faecium*, we conducted a comprehensive RNA sequencing (RNAseq) transcriptome analysis on the *E*. *faecium* vancomycin resistant strain Aus0004 isolated from a bloodstream infection^14^. Transcription profiles during the incubation of the bacteria inside the mice peritoneum were compared to the transcriptome of bacteria grown in standard laboratory conditions. Among the *in vivo* induced transcripts were four LysM domain-containing proteins, EFAU004_01209, EFAU004_01059, EFAU004_01150 and EFAU004_00494. In a recent study, we highlighted, among others, a LysM domain-containing protein as a promising vaccine candidate in *E. faecium*^15^. The LysM domain is a carbohydrate-binding module conserved across all kingdoms^16^. It is mostly present in bacterial extracellular proteins, including peptidoglycan (PG) hydrolases, adhesins and virulence factors^17^. Prokaryotic LysM modules bind non covalently to the N-acetylglucosamine moieties of PG, the main component of the bacterial cell wall^18^. This domain can be present in one or multiple copies and be located at the N- or C-terminal parts of the corresponding proteins^18^. On the basis of the induction of the corresponding genes, we decided to study the physiologic *in vitro* and *in vivo* roles of the LysM domain-containing proteins in *E. faecium* AUS0004.

## Results and Discussion

### Interpretation of the infection transcriptome of *E. faecium*

To gain an understanding of the adaptation of *E. faecium* to the host environment and identify cell wall associated proteins expressed during infection, *E. faecium* strain Aus0004^14^ was-inoculated into the mouse peritoneum, and bacteria were recovered 24 hours post-infection. In parallel, bacteria were grown *in vitro* in brain heart infusion broth (BHI) to the exponential mid log phase (O.D._600_ = 0.4) and stationary growth phase (24 hour culture). Total RNA from bacteria grown *in vivo* and *in vitro* was extracted, and RNAseq was performed to analyze the transcriptional activity under these different conditions. Considering a threshold fold change of 2 as significantly differentially expressed, we obtained 715 and 613 overexpressed genes in the analysis against the stationary phase and exponential phase cultures, respectively. Since the physiological state of the bacteria during the infection was unknown, we decided to consider only the overlapping genes between the two analyses, reducing our *in vivo* database to 362 genes. The list of genes identified as upregulated in both conditions is shown in Supplementary Table S4. A comparable study has been previously conducted on *E. faecalis*^19^. The main conclusions that can be drawn from the transcriptome data for both bacteria species is that the strategy *E. faecium* uses to adapt to host conditions is profoundly different from the strategy of *E. faecalis*. Only two orthologous, *in vivo* induced operons, overlap between the transcriptomes of the two species; two *E. faecium* paralogous genes, EFAU004_00919 and 01244, overlap with EF0202 of *E. faecalis*, and EFAU004_00560-64 of *E. faecium* overlaps with EF02390-94 of *E. faecalis*. The EFAU004_00919, EFAU004_01244, and EF0202 genes encode phosphopyrimidine kinases converting hydroxymethylpyrimidine monophosphate HMP~P with ATP as the co-factor to HMP~P~P. In *E. coli*, this reaction is part of the biosynthesis-pathway of Thiamine~P~P^20^. The loci EFAU004_02390-02394/EF0560-0564 correspond to the sufCDSUB operon in both species, which is involved in the assembly of Fe-S clusters in corresponding proteins^21^. While *E. faecalis* induced numerous genes encoding stress defense proteins^19^, only four stress genes are induced in *E. faecium*. Two of them encode peptide methionine sulfoxide reductases (Msr) (EFAU004_00288, EFAU004_01188). Methionine (Met) is among the most sensitive amino acids to oxidative damage and is converted into methionine sulfoxide (MetSO). Msr are enzymes that reduce MetSO back to Met^22^. The genome of *E. faecium* AUS0004 strain harbors five genes encoding these activities, and the induction of two of them might indicate that the bacteria were submitted to mild oxidative stress. A further hint that the bacteria were stressed in the peritoneum is the strong induction of two genes encoding the substrate binding domain and transporter ATP binding subunit of a glycine betaine transport system. This system catalyzes the accumulation of glycine betaine, a compatible solute that alleviates the effects of osmotic stress^23^. A further striking difference between the enterococcal species relates to the induction of ABC transporters. Considerably more of these transporters were induced in *E. faecium* (N° = 14) than *E. faecalis* (N° = 5). Several of these operons were annotated as amino acid or peptide ABC transporters, and some of them were very strongly induced (>120-fold). The latter observation may be related to the induction of numerous genes encoding peptidases (N° = 6) and seems to suggest the bacteria have an increased need for the supply of amino acids inside the mouse peritoneum. This is further supported by the strong induction (nearly 200 fold) of a gene annotated as amino acid permease (EFAU004_00626). The data also seem to reflect that *E. faecium* divides in the peritoneum, since genes encoding key cell division proteins (FtsA: EFAU004_00770, FtsZ: EFAU004_0771, DivIVA: EFAU004_00994, and FtsK: EFAU004_02286) were induced under the *in vivo* conditions. We tried to verify cell growth in the peritoneum. However, CFU counts did not change much over time at 24 h post infection as shown in Fig. S2. This may reflect a balance between cells phagocytized by polymorphonuclear cells, bacteria that translocate into organs and cells dividing in the peritoneum. To proliferate, the bacteria had to find nutrients, including an energy source, inside the peritoneum. The transcriptome database of *E. faecalis* highlighted several putative substrates, including glycerol and maltodextrines^19^. In *E. faecium*, glycerol-3-phosphate (glycerol-3-P) is a potential substrate, since a glycerophosphodiester phosphodiesterase (EFAO004_01774) was strongly induced in the peritoneum. This enzyme is implicated in glycerophospholipid metabolism and the hydrolysis of glycerophosphodiester liberates glycerol-3-P. The resulting catabolic enzyme, glycerol-3-P dehydrogenase, generates dihydroxyacetone phosphate, which enters the glycolytic pathway. Interestingly, a gene (EFAU004_02253) annotated to encode this final activity was also significantly induced during peritonitis. Another substrate for the growth of *E. faecium* inside the mouse peritoneum might be ascorbate, since the corresponding operon encoding enzymes implicated in the transport (EFAU004_01270-72, EFAU004_01296) and conversion (EFAU004_1267, 1269, 1273) of ascorbate to D-xylulose-5-phosphate were significantly induced. It has been recently shown that *E. faecalis* can grow on ascorbate as the sole energy source^24^, and this might also be the case for *E. faecium*. The *E. faecium* strain AUS0004 is a vancomycin resistant clinical isolate that harbors a chromosomal *vanB* operon^14^ whose expression is induced by the presence of the glycopeptide^25^. However, other stimuli seem to induce expression of the *van* genes, since the entire operon (EFAU004_02774-79) was highly induced (~16-fold) in bacteria present in the mouse peritoneum in the absence of the antibiotic. The significance of the induction of the van operon for the infection process of *E. faecium* remains unknown. A substantial number of the *in vivo* induced genes are implicated in peptidoglycan synthesis and modification. The most important differences in expression concern four penicillin binding or penicillin binding domain protein encoding genes (EFAU004_00870, 00997, 01299, 02553) which were induced between 3 and 25 fold in the mouse peritoneum. The *dlt* operon (EFAU004_00725-28) was highly induced. It encodes proteins implicated in the addition of D-alanine to teichoic acids of the cell wall, which results in the reduction of the net anionic charge of the bacterial cell envelope^26^. This D-alanylation of cell wall polymers increases the resistance of bacteria to antimicrobial peptides^26^, which are potentially encountered in the peritoneum. Interestingly, among the most highly *in vivo* induced genes are four *E. faecium* genes encoding LysM domain proteins (EFAU004_00494, 01059, 01150, 01209), which show induction factors ranging from 4 fold to 600 fold (Table S4). This might indicate that the corresponding proteins, especially those that are highly induced, are important for the pathogenicity of *E. faecium*. Therefore, we decided to study their roles in the infection process in more detail.

### General characteristics of the LysM domain-containing proteins of *E. faecium*

**Figure 1:**
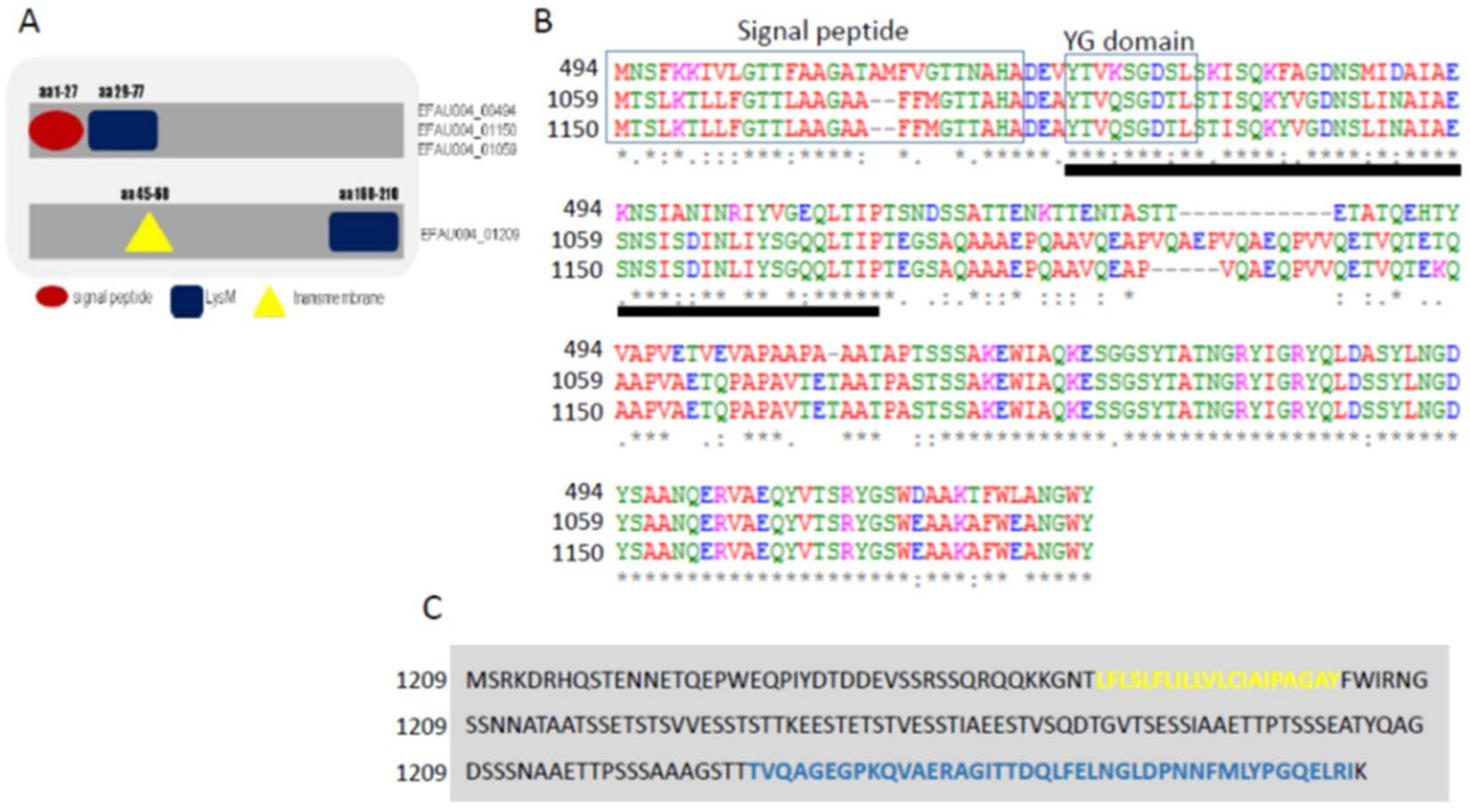
Domain organization and alignment of *E. faecium* LysM domain–containing proteins. (**A**) Domain organization of the LysM-containing proteins. EFAU004_00494, EFAU004_01150 and EFAU004_01059 have the same organization, whereas EFAU004_01209 is organized differently. Red circle: signal peptide; blue square: LysM domain; yellow triangle: transmembrane domain. (**B**) Clustal Omega alignment of proteins EFAU004_00494, EFAU004_01150 and EFAU004_01059. The signal peptide and the conserved YG domain are boxed. The LysM domain is indicated by the black line below the alignment. (**C**) Sequence of the EFAU004_01209 LysM protein. The transmembrane helix and the LysM motif are highlighted in yellow and blue, respectively.

The genes encoding the four LysM domain-containing proteins are very likely organized in monocistronic operons. Compared to EFAU004_1209, which was significantly expressed under laboratory conditions and slightly induced *in vivo*, the genes encoding the three other LysM proteins were weakly expressed *in vitro* but highly induced *in vivo*. The count per million reads (cpm) of the corresponding genes are shown in Supplementary Table S5. To confirm the RNAseq data, we performed a real-time quantitative PCR on EFAU004_01209 gene in the three different conditions described before. The data, shown in Fig. S1, confirmed the RNAseq analysis. The LysM proteins are comparable in size and have isoelectric points ranging from 202 AA to 212 AA and 4.24 to 4.93. Three genes (EFAU004_00494, 01059 and 01150) were annotated as encoding the endopeptidase M23, although the characteristic HxH motif^27^ is absent in these proteins. The three proteins are highly homologous, especially proteins EFAU004_01059 and EFAU004_01150, which differ only by an additional sequence (VQAEP) present nearly in the middle of protein EFAU004_01059 (Fig. 1B). All of the proteins contain a signal sequence at the N terminus followed by the LysM domain harboring the highly conserved YG motif (Fig. 1A). No significant homology exists between the abovementioned proteins and EFAU004_01209 (Fig. 1C). Its LysM domain, situated at the C-terminal part of the protein, is more distant from the consensus sequence, even in the well conserved YG motif. Furthermore, a signal sequence is absent in this protein, but it contains one transmembrane helix (Fig. 1A,C). A Blast analysis with the amino acid sequences of the LysM domain-containing proteins as the input revealed the presence of 97% and 100% conserved orthologues of EFAU004_01150 and EFAU004_01209 respectively, of unknown function in a particular *Clostridioides difficile* strain (strain Y384).

### Construction and characterization of lysM deletion mutants

**Figure 2:**
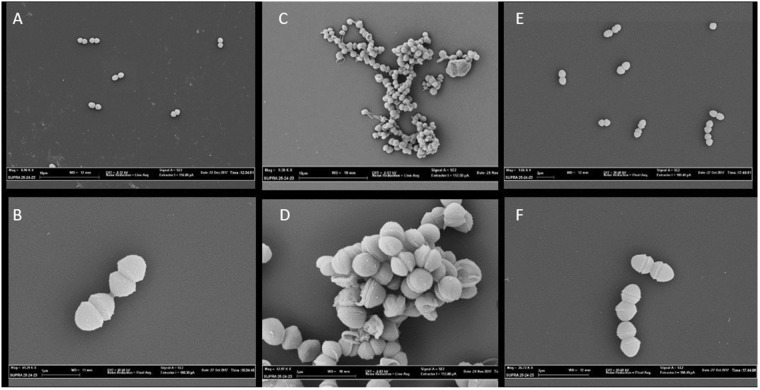
Scanning electron microscopy images of: (**A**,**B**) AUS0004. (**C**,**D**) ΔEFAU004_01209 deletion mutant. (**E** and **F**) Δ1209:1209 complemented strain. (**A**,**C**) bar = 10 µm. (**B**) bar = 1 µm (**D**,**E**,**F**) bar = 2 µm.

**Figure 3:**
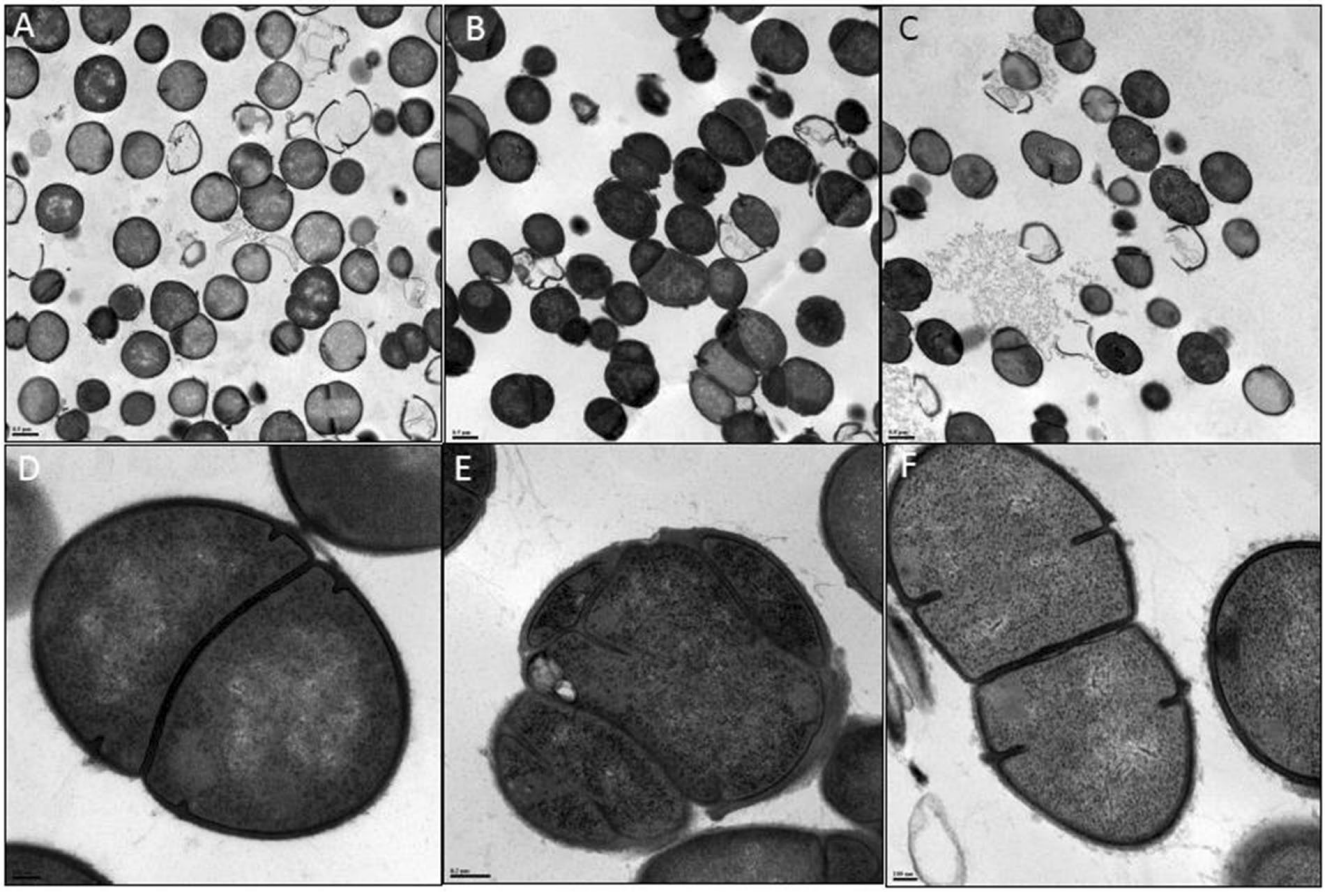
Transmission electron microscopy images of: (**A**,**D**) AUS0004; (**B**,**E**) ΔEFAU004_01209 deletion mutant, (**C**,**F**) Δ1209:1209 complemented mutant. (**A**–**C**) bar = 0. 5 µm. (**D**,**F**) bar = 0.1 µm. (**E**) bar = 0.2 nm.

To evaluate the role of the LysM domain-containing proteins in the *E. faecium* infection process, deletion mutants of EFAU004_00494, EFAU004_01059, EFAU004_01150 and EFAU004_01209 were constructed. Due to the high similarity of EFAU004_01059, EFAU004_01150 and EFAU004_00494 and their therefore expected redundant cellular roles, we also constructed the double mutant ∆EFAU004_01059-01150 and the triple mutant ∆EFAU004_01059-01150-494. The growth rates of all mutant strains in laboratory medium was comparable to that of the parent strain, as shown in supplementary Fig. S3. However, an initial slight decrease of OD was observed for the ∆EFAU004_01209 and we noticed that the mutant EFAU004_01209 had the tendency to sediment to the bottom of the test tube, while the wildtype, complemented and other mutant strains grew normally, resulting in uniform turbid medium with few sedimenting cells. (Fig. S4, supplemental materials). Subsequent cell morphology studies by scanning electron microscopy (SEM) showed that, compared to the wild type, the Δ1209 mutant had an irregular morphology with unseparated daughter cells, suggesting a defect of cell separation (Fig. 2). Transmission electron microscopy (TEM) observations further supported the inhibition of cell separation in the mutant cells (Fig. 3). The mutant cells showed anarchic septa formations leading to emergence of mini compartments inside the cells. These alterations were restored in the complemented strain.

### Implication of LysM domain-containing proteins in *E. faecium* virulence

**Figure 4:**
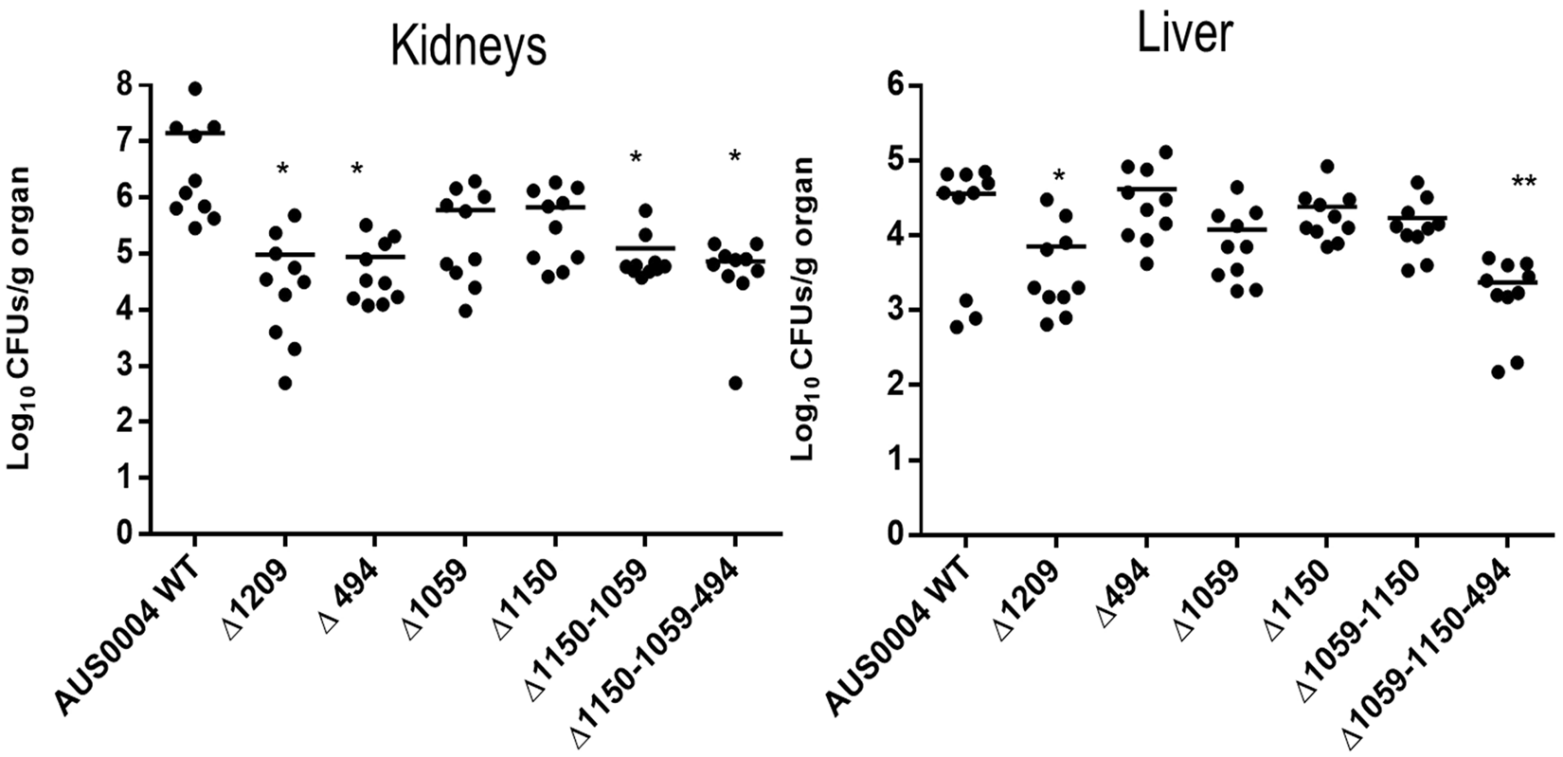
Effect of AUS0004 (wild-type strains) and the deletion mutants in a mouse model of systemic infection. Groups of 10 BALB/c female mice were injected intravenously with approximately 1 × 10^8^ cells of the indicated strains. Data are expressed as the log_10_ colony-forming units (CFUs)/g of bacteria recovered from kidney and liver homogenates seven days after the challenge. The log_10_ CFUs of both kidneys were combined and averaged. A value of 0 was assigned to the uninfected organs. Horizontal bars represent geometric means. Log_10_ counts were compared for statistical significance by two-way ANOVA. P values < 0.05 were considered significant. ^*^<0.05, ^**^<0.005, ^***^<0.001.

**Figure 5:**
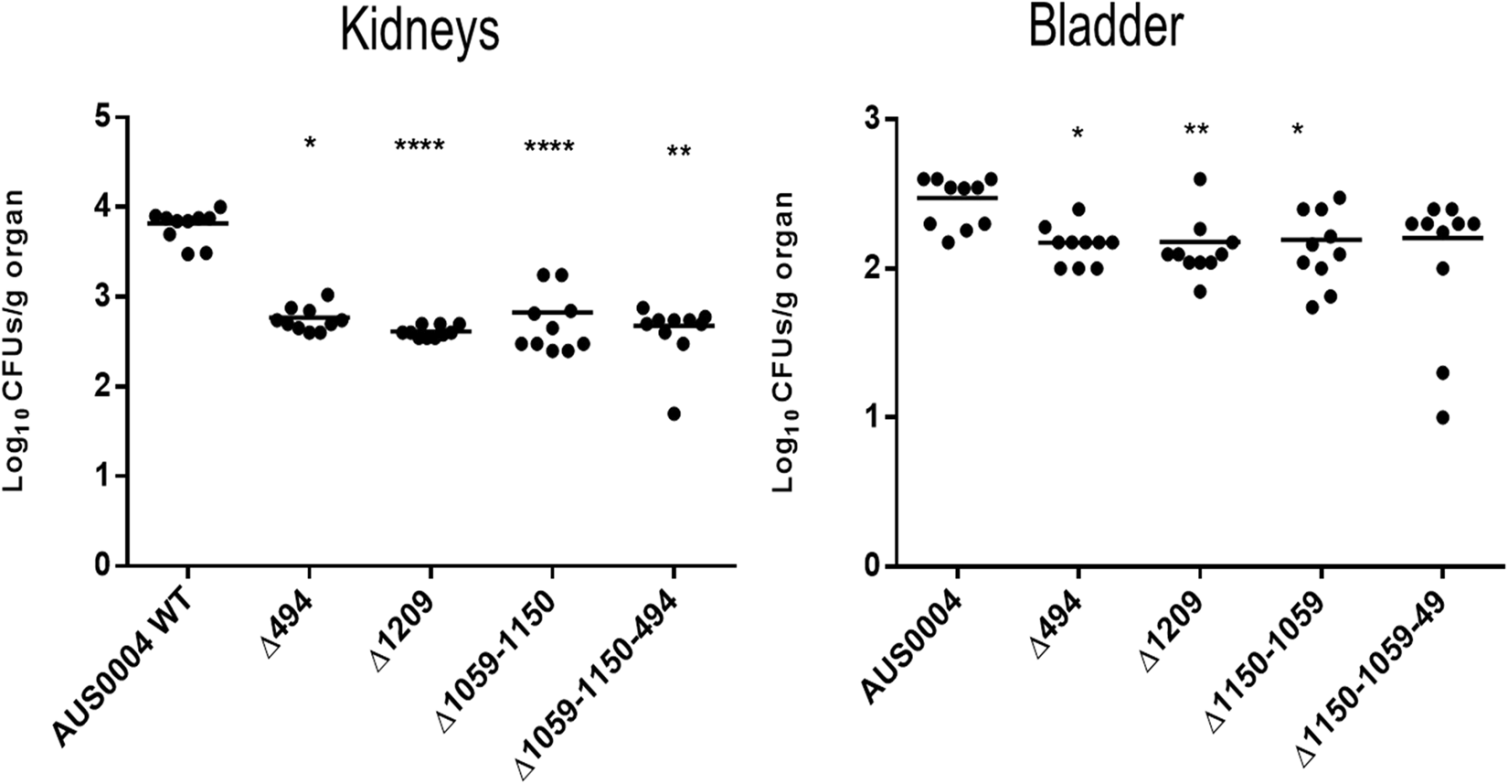
Effect of AUS0004 (wild-type strains) and the deletion mutants in a mouse model of urinary tract infection. Groups of 10 BALB/c female mice were transurethral challenged with approximately 1 × 10^8^ cells of the indicated strains. Data are expressed as the log_10_ colony-forming units (CFUs)/g of bacteria recovered from kidney and urinary bladder homogenates four days after the challenge. The log_10_ CFUs of both kidneys were combined and averaged. A value of 0 was assigned to the uninfected organs. Horizontal bars represent geometric means. Log_10_ counts were compared for statistical significance by two-way ANOVA. P values < 0.05 were considered significant. ^*^<0.05, ^**^<0.005, ^***^<0.001.

Since the LysM encoding genes were overexpressed during the infection, we postulated that the corresponding proteins might play important roles during infection. We used two different animal model approaches, systemic and urinary tract infections, to detect a possible decrease in the colonization ability of the mutants compared to the wild type. The wild type and the isogenic mutant strains were injected intravenously in Balb/C mice. Seven days post infection, we examined the tissue burdens in the kidneys and livers of the infected mice. As shown in Fig. 4, the single mutants EFAU004_01059 and EFAU004_01150 did not exhibit tissue burden differences compared to the wild type. Conversely, the single mutants ΔEFAU004_00494 and ΔEFAU004_01209 and the double ∆EFAU004_01059-01150 and the ∆EFAU004_01059-01150-494 triple mutants showed a significantly decreased colonization in the kidneys. The AUS0004 wild type strain and the mutants displayed a low tropism for liver. The ΔEFAU004_01209 strain and the triple mutant ∆EFAU004_01059-01150-494 showed a decreased tissue burden. Since enterococci can cause urinary tract infections, and the *E. faecium* AUS0004 strain showed high tropism for kidneys in the systemic model, we used a mouse model of urinary tract infection to assess the effect of the deletions. Female Balb/C mice were infected via intra urethral catheterization, and, 4 days after infection, their bladders and kidneys were homogenized and serial dilution plated to enumerate the CFU per gram of organ. As shown in Fig. 5, the numbers of bacteria recovered from the single mutants ∆EFAU004_1209 and ∆EFAU004_00494, as well as from the double and the triple mutants, were significantly lower than those of the wild type and the other mutant strains, in accordance with the phenotype detected in the systemic model.

### Cell adhesion assay

**Figure 6:**
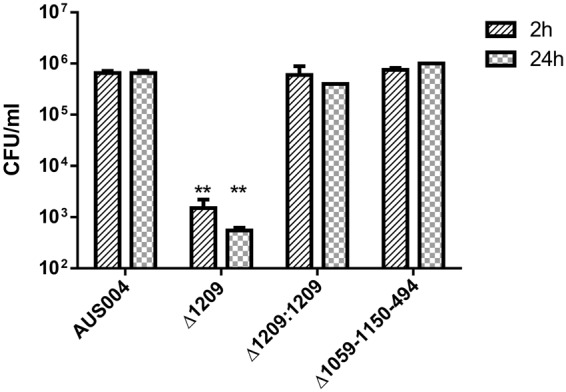
Adherence of *E. faecium* AUS0004 wild type and its isogenic mutant strains, the triple mutant ∆EFAU004_01059-01150-494 and the single ΔEFAU004_01209 mutant, and the complemented strain Δ1209:1209 to kidney epithelial VERO cells after two and twenty-four hours. Cells were infected with an MOI of 100:1. Adherent bacteria were collected after 2 and 24 hours and enumerated by plating serial dilutions on BHI agar plates and counting CFU. The bars indicate standard deviations. The assay was performed in triplicate and repeated twice. The analysis was performed by two-way ANOVA. p values < 0.01 were considered significant. ^**^p = 0.001.

A possible contribution of the LysM domain-containing proteins in the *E. faecium* adherence process was tested on kidney epithelial cells, as previously described^28^. VERO cells were infected with an MOI of 100:1 with the AUS0004 wild type, the triple mutant ∆EFAU004_01059-01150-494, the ΔEFAU004_01209 single mutant strains and the corresponding complemented strain Δ1209:1209. Samples were collected at 2 and 24 hours post infection, and the numbers of adhered cells were enumerated. As shown in Fig. 6, the adherence of the triple mutant was comparable to that of the parent strain. In contrast, the adherence of the ΔEFAU004_01209 mutant was significantly reduced compared to that of the wild type. The decreased ability of the ΔEFAU004_01209 mutant to adhere to kidney epithelial cells could explain its decreased ability to colonize mice kidneys during infection, although we cannot exclude that the reduced adherence of the ∆EFAU004_01209 mutant could be due to the defect of correct cell separation and tendency to aggregation. Both phenotypes observed in the ΔEFAU004_01209 mutant could in fact indirectly interfere with the adhesion to the cell monolayer.

### LysM domain containing protein EFAU004_01209 is a surface exposed protein

**Figure 7:**
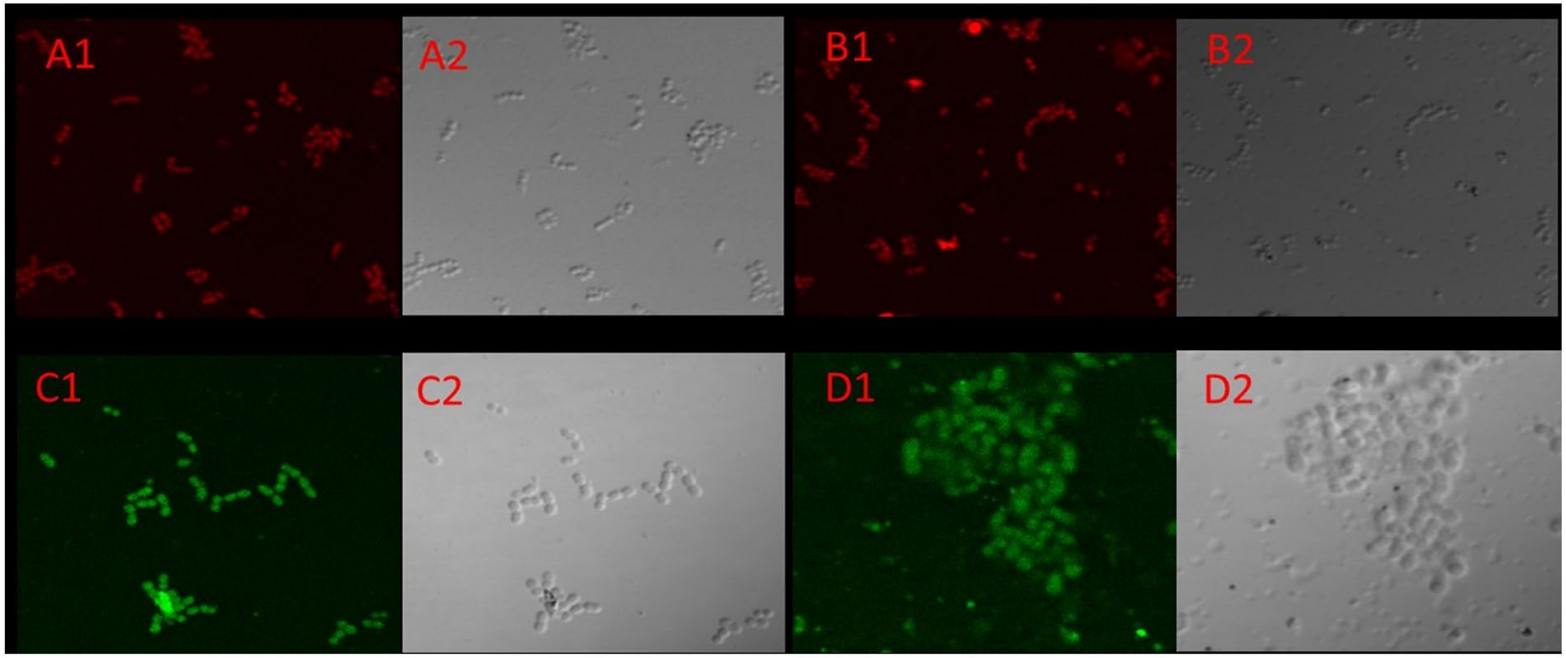
Immunofluorescence microscopy of the enterococcal strains labeled with anti-1209 serum coupled with fluorescein isothiocyanate (FITC) (green); cells are stained with Evans blue. (**A**) A1 ΔEFAU004_01209. No signal is detected. A2 relative phase channel (**B**) B1 Anti-CcpA serum on total protein extracts of the *E. faecalis* JH2-2 strain; no immunofluorescence signal is detected due to the cytoplasmic localization of the CcpA protein. B2 Relative phase channel (**C**) C1 AUS0004 wild-type strain shows green fluorescence, indicating a possible surface localization of the EFAU004_1209 protein. C2 relative phase channel (**D**) D1 Triple mutant Δ1050-1150-494. D2 Relative phase channel.

Unlike the other three highly homologous LysM containing proteins, the *EFAU004_01209* protein has no conventional leader sequence required for protein export via the general secretory pathway, but it shows a transmembrane helix motive in its N-terminal part. Furthermore, the protein appears to have a role in septum formation during cell division. This set of elements has led us to hypothesize a probable localization of this protein on the surface of the bacterium. To demonstrate that the *EFAU004_01209* protein is a surface exposed protein, we overexpressed and purified recombinant rEFAU0004_01209, and mice were subcutaneously immunized to obtain the corresponding polyclonal anti- EFAU004_01209 serum. The specificity of the serum is shown in Supplementary Fig. S5. The localization of EFAU004_01209 was determined by immunofluorescence experiments on whole cells of the wild-type strain AUS0004 and the triple mutant strain ΔEFAU004_01150-1059-494 using the specific mouse antiserum. A uniform fluorescence distribution around the bacterial cell surface was observed, indicating that the *EFAU004_01209* protein was present on the cell surface of the *E. faecium* strain AUS0004 (Fig. 7C1,D1). We further tested an anti-CcpA serum that recognizes the cytoplasmic protein CcpA^29^. No signal was detected (Fig. 7B1), demonstrating that, under our experimental conditions, cytoplasmic proteins were not detected. In addition, no fluorescence signal is detected in the ΔEFAU004_01209 strain, confirming the specificity of the signal (Fig. 7A1).

### LysM domain containing protein EFAU004_01209 might be a promising target for treatment of UTI

**Figure 8:**
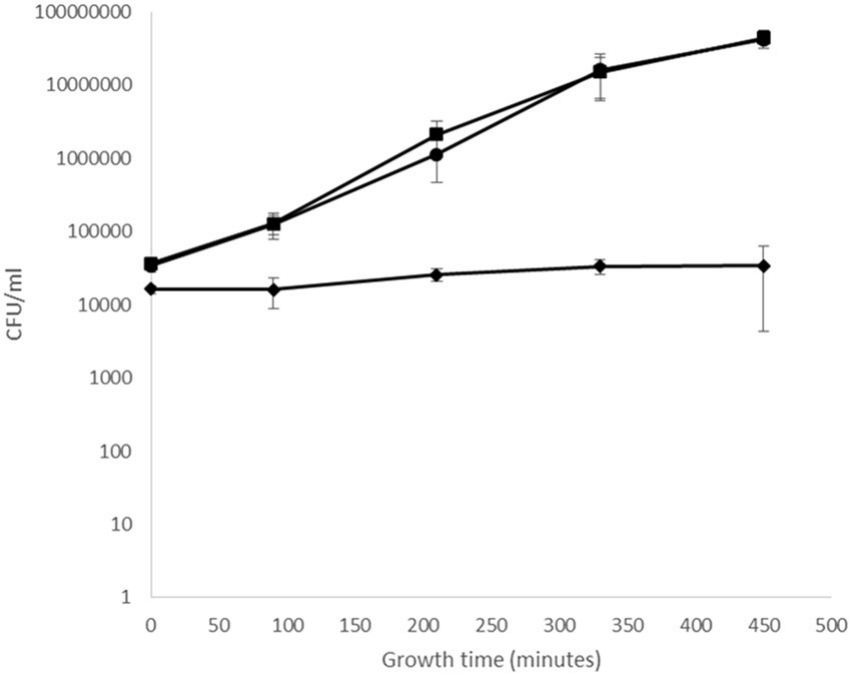
Growth in human urine. Bacteria were inoculated in filtered urine and incubated for 7.5 hours at 37 °C, and CFU/ml counts determined for the wild-type strain (black square), the ∆1209:1209 complemented strain (black circle) and ∆EFAU00_01209 mutant strain (black rhombus). The assay was performed in duplicate. Error bars represent the standard deviations.

Enterococci are leading causes of urinary tract infections-(UTI)^30^.Treatment has increasingly been challenging because of their intrinsic and acquired resistances to recently introduced antibiotics^31^, and vancomycin resistant enterococci (VRE), along with multiply resistant enterococcus (MRE) strains, are now common in UTI^32^. We tested the wild type and mutant strains for growth in human urine and found that the growth of the ΔEFAU004_01209 mutant was strongly affected in this bio fluid, whereas its growth in laboratory medium is comparable to that of the parental strain (Fig. 8). Wild type growth was restored in the complemented strain. This interesting finding may identify the peptidoglycan associated LysM domain-containing protein encoded by the EFAU004_01209 gene as an attractive new drug target for the treatment of UTI caused by multiple resistant *E.faecium* strains.

## Conclusions

In conclusion, this work presents for the first time a global transcriptomic analysis of the adaptation of the health care associated pathogen *E. faecium* to an infection site. This allowed the establishment of a database of *in vivo* induced genes. We focused our efforts on the four genes encoding the LysM domain containing proteins of *E. faecium*, which were significantly induced under infection conditions. These proteins had unknown cellular functions. The most interesting phenotypes were obtained with the ∆EFAU004_01209 mutant. The corresponding gene was significantly expressed both *in vitro* during growth in brain hearth infusion broth and *in vivo* during the intra peritoneal infection, suggesting that it has an important cellular role. The corresponding protein is surface exposed and indispensable for proper cell division, suggesting its role in cell wall synthesis. The observed morphological modifications are very comparable to those observed for a *sle1* mutant of *Staphylococcus aureus*^33^. These authors showed that the protein encoded by the *sle1* gene had *N*-acetylmuramyl-L-alanine amidase activity. Despite the fact that the EFAU004_01209 gene showed no homology with *sle1*, we conducted a zymographic analysis, as described in the work of Kajimura and collaborators^33^. However, no lytic activity in the *Micrococcus luteus* gel was detected for the EFAU004_01209 recombinant protein. Work is in progress to define the exact role of the EFAU004_01209 protein in peptidoglycan synthesis. Altogether, the surface exposed protein EFAU004_01209 could correspond to a new interesting drug target since the absence of this protein reduces significantly the capacity of host colonization. Regarding the other three LysM domain-containing proteins, EFAU004_01059, EFAU004_01150 and EFAU004_00494 were found to be highly similar, especially EFAU004_01059 and EFAU004_01150. In the systemic and urinary tract infection models, only the infection with the double and the triple mutants resulted in a significantly decreased tissue burden compared to the wild type, while the only single mutant with a reduced organ colonization ability was EFAU004_00494. This led us to speculate that EFAU004_00494 may have a specific role unrelated to the other two enterococcal proteins, which seem to have redundant roles. Blast analysis revealed a 56% homology with the SS9 LysM domain protein of *Streptococcus suis*, characterized as involved in the virulence of the bacterium; experiments conducted on the SS9 deletion mutant demonstrated that the mutant was more sensitive to the antibacterial activity of host macrophages *ex vivo*. The concentration of free iron in the blood was lower in mice infected with the SS9 mutant compared to the wild type, suggesting that the SS9 protein contributes to virulence by releasing more free iron in the blood of the host^34^. A similar role may be attributed to the three LysM domain-containing proteins, which are also supposed to be secreted outside the cell. Future experiments on survival inside the host will be conducted to verify this hypothesis.

## Material and Methods

### Bacterial strains and growth media

The *E. faecium* strains indicated in Supplementary Table S1 were grown at 37 °C in Brain Heart Infusion (BHI, Sigma Aldrich, Saint Louis, MO, USA). To grow the cells on solid media, BHI agar was used. When required, spectinomycin (Sigma Aldrich) at a final concentration of 500 μg/ml was added. The *Escherichia coli* strains (Supplementary Table S1) EC1000, Top10 and BL21 were grown in liquid and solid Luria Bertani (LB Sigma Aldrich) media. If necessary, kanamycin (50 μg/ml) (Sigma Aldrich) or spectinomycin (100 μg/ml) was added to the medium. Human urine was collected from a healthy volunteer who had no history of UTI or antibiotic use in the last 6 months. The urine was centrifuged at 12000 × g and sterilized by filtration (0.22 µm pore size). Since the composition of human urine may be variable, samples were collected on three separate days for three replicate experiments and used by the next day.

### Transmission electron microscopy

The cells were rinsed in physiological water and fixed (2.5% glutaraldehyde in 0.1 M RR cacodylate buffer at pH 7.0 containing 0.04% ruthenium red) for 15 hours at 4 °C. The cells were then rinsed in RR cacodylate buffer, post fixed 1 hour with 1% osmium tetroxide in RR cacodylate buffer (at 4 °C protected from light), rinsed in RR cacodylate buffer, pelleted in 1.5% agar with a low melting point of 40 °C, dehydrated in progressive bath of ethanol (70–100%), embedded in resin (Embed 812) and polymerized for 24 h at 60 °C. Ultrathin sections were prepared and compared with uranyl acetate and lead citrate. The cells were observed with the JEOL 1011 transmission electron microscope and images were taken with the ORIUS 200 camera and digital micrograph software.

### Scanning electron microscopy

The cells were rinsed in physiological water, fixed with 2.5% glutaraldehyde in 0.1 M cacodylate buffer at pH 7.0 for 15 hours at 4 °C, rinsed in 0.1 M cacodylate buffer at pH 7.0 and sedimented for one week on Thermanox® coverslip coated with poly-L-lysine. The cells were then dehydrated in a progressive bath of ethanol (70–100%) and critical point dried (CPD 030 LEICA Microsystem). The cells were sputtered with platinum and observed with the JEOL 6400F scanning electron microscope.

### RNA purification

Total RNA was extracted from the *E. faecium* Aus0004 strain cultured in BHI (Brain Heart Infusion Medium) during the mid-exponential (OD_600 nm_ = 0.3) or stationary growth phase (24 h of culture) and from bacteria recovered from mice peritoneum after infection. Pellets were resuspended in 200 µl of Max Bacterial Enhancement Reagent (Thermo Scientific, Breda, the Netherlands). This suspension was added to a 2:1 volume of acid phenol (Thermo Scientific) with glass beads and then agitated for 30 min at 30 Hz in the MM200 shaker (Retsch GmbH, Haan, Germany). After 20 minutes of centrifugation, the aqueous phase was taken and transferred to 1 volume of TRIZol (Thermo Scientific), mixed and incubated for 5 min at room temperature. One volume of ethanol was then added. RNA was then purified using Direct Zol RNA Miniprep (Zymo Research, Irvine, USA CA) according to the manufacturer’s instructions.

### RNA sequencing

The RNA sequencing experiments were performed at the iGE3 genomics platform of the University of Geneva. A total of 1 µg of total RNA was ribo depleted using the Ribo Zero Magnetic Kit for bacteria from Epicentre (Illumina, Wisconsin, USA). Libraries were then prepared using the Illumina TruSeq stranded mRNA kit according to manufacturer’s recommendations. Libraries were validated on the Bioanalyzer 2100 (Agilent Technologies, Santa Clara, USA) and the Qubit fluorimeter (Thermo Scientific). Samples were multiplexed by 8 and loaded at 8 pM on one lane of an Illumina HiSeq 2500 according to the single read, 50 cycle protocol.

### Bioinformatic analysis

The sequencing quality control was done with FastQC^35^. The reads were mapped with the TopHat v.2 software to the *Enterococcus faecium* Aus0004 reference^14,36^. The table of counts with the number of reads mapping to each gene feature of the Aus0004 reference genome was prepared with HTSeq v0.6p1 (htseq count). The differential expression analysis was performed with the R/Bioconductor statistical analysis package EdgeR v. 3.4.23^37^. The counts were normalized according to the library size and filtered. The genes with a count above 1 count per million reads (cpm) in at least 2 samples were kept for further analysis. The differentially expressed gene tests were statistically assed with the exact test in EdgeR. The differentially expressed gene p-values were corrected for multiple testing error with a 5% FDR (false discovery rate). The correction used was Benjamini Hochberg (BH).

### Mutants construction

To generate deletion mutants for the EFAU004_00494, EFAU004_01059, EFAU004_01150 and EFAU004_01209 genes, we used the thermosensitive PWS3 vector. The plasmids used in this study are listened in Supplementary Table S2^38^. Regions 1 kb upstream and downstream of the genes of interest were amplified by the Kapa HiFi PCR mix (Kapa Biosystem, Wilmington, Massachusetts, USA) using the primers listed in Supplementary Table S3. The purified PCR products were used as a template for overlapping PCR (primers FW2 and RV5, Table S3). The samples were then digested with the restriction enzymes (New England Biolabs) indicated in Table S3 and ligated in PWS3. The resulting vectors were then inserted into the *E. faecium* Aus0004 genome by electrotransformation followed by homologue recombination and excision, as previously described^38,39^. The effective deletion was confirmed by PCR and the sequencing of regions of interest. For complementation, the entire EFAU004_01209 gene with its promoter and the regions between 1597888–1598925 (genomic coordinates) and 1598926–1599972 were amplified using the Kapa HiFi PCR mix (Kapa Biosystem, Wilmington, Massachusetts, USA) using the primers listed in Table S3. The purified products were used for overlapping PCR (primers OCG119 and OCG124). The resulting products were digested with SacII and SpeI (NEB) and cloned into the vector pWS3^39^. The vector was electroporated into the ∆1209 strain to obtain the single copy chromosomal *trans-*complemented strain, following the protocol described for the construction of the mutant.

### Expression of recombinant EFAU004_01209

The EFAU004_01209 PCR purified product was cloned into the pETSumo vector (Thermo Scientific) according to the manufacturer’s instructions. The expression and purification of the recombinant EFAU004_01209 (rEFAU004_01209) under native conditions was performed as described previously^40^. The eluted rEFAU004_01209 protein was dialyzed against PBS (phosphate buffered saline).

### Bacterial infection of mice

For *in vivo* RNA preparation, the enterococcal cells were grown overnight and then pelleted and resuspended in 5 ml of phosphate buffered saline (PBS) for injection. Mice were infected by intraperitoneal injection of 1000 μl, corresponding 10^7^ to 10^8^ cells. Twenty four hours after inoculation, the bacteria and eukaryotic cells were collected by peritoneal washing. The eukaryotic cells were then lysed in ice cold water for 45 minutes. The bacteria were resuspended in PBS, and serial dilutions were made on TSA medium to quantify recovery. For the intravenous infection model, overnight cultures of the strains were centrifuged, and pellets were resuspended in PBS at final concentrations of 10^9^ bacteria/ml. A total of 100 μl from each strain was injected into 10 female BALB/c mice (10 weeks old; Harlan Italy S.r.l., Udine, Italy) through the tail vein. Mice were euthanized by cervical dislocation 7 days after infection. Kidneys and livers were then removed, weighed, and homogenized using a stomacher (model 80; Pbi International, Milan, Italy). Serial homogenate dilutions were plated onto enterococcus selective agar (Fluka Analytical, Switzerland) for CFU determination. The CFU counts were analyzed by the unpaired *t*-test. Overnight grown strains were centrifuged, and pellets were resuspended in PBS to achieve final concentrations of 10^9^ C.F.U./ml. Ten six weeks old female mice were infected via intraurethral catheterization (polyethylene catheter, 4 cm long; outside diameter, 0.61 mm; Becton Dickinson, Sparks, MD) with 100 μl of each strain suspension. Mice were sacrificed four days after transurethral challenge, and the bladders and kidneys were excised, weighed, and homogenized with a Stomacher 80 device (PBI International, Italy) for 120 s at a high speed. Serial homogenate dilutions were plated for CFU determination. The CFU counts were analyzed by the unpaired *t*-test.

### Production of polyclonal antiserum

To obtain an anti EFAU004_1209 serum, two male BALB/c mice (6 to 8 weeks old; 25 to 30 g) were immunized with recombinant purified rEFAU004_1209. Animals were immunized subcutaneously with 25 μg of rEFAU004_1209 in Freund’s complete adjuvant, and then two boosters containing the same amount of protein/adjuvant were given at 3 week intervals. Three weeks after the final inoculation, mice were injected intraperitoneally with 10 μg of rEFAU004_1209 mixed with uncomplete Freund’s adjuvant. Animals were bled 2 weeks after each booster, and sera were tested for anti rEFAU004_1209 antibody titer by immunoblotting.

### Real-time quantitative PCR

To test EFAU004_01209 expression levels in AUS0004, RNA was extracted from bacteria grown in BHI medium till exponential phase (O.D.: 0,4) and stationary phase culture (24 hours) and from bacteria recovered after intra-peritoneal infection, as described before, using RNeasy mini kit (Qiagen), which includes an RNase free DNase treatment step to eliminate DNA. Quantitative real-time reverse transcription PCR (RT-PCR) was performed in a CFX96 system (BIO Rad Laboratories), using *gyrA* as normalization gene^19^using primers listed in supplementary Table S3. The relative messenger RNA (mRNA) expression level of the target gene in each sample was calculated using the comparative cycle threshold method.

### Immunofluorescence

For the immunofluorescence staining, whole cells of the AUS004 wild type strain, ΔEFAU004_1209 and the triple mutant strains, grown overnight, were fixed with PBS containing 4% paraformaldehyde for 5 minutes and then incubated overnight with a polyclonal antiserum against rEFAU004_1209 that was generated in mouse. The bound antibodies were detected by incubation for 1 hour with fluorescein isothiocyanate (FITC) coupled anti mouse antibodies (Sigma). A total of 15 µl of the sample was applied to a glass slide, air dried, and heat fixed. The analysis was performed with a confocal microscope (Eclipse Ti-E Nikon). Pre-immune antiserum from the same animal was used as a negative control. In the same experiments, the same strains were also considered without the incubation with the primary antibody.

### Cell adhesion assay

Kidney epithelial cells from monkey (VERO cells) were cultured in Dulbecco’s modified Eagle medium (DMEM) (Gibco, Invitrogen, United Kingdom) supplemented with 10% heat inactivated fetal calf serum (Integro B.V., Zaandam, The Netherlands), 1% nonessential amino acids (Gibco), and 1 mM glutamine (Gibco) and incubated in a 37 °C incubator with 5% CO_2_. Differentiated VERO cells were prepared by seeding cells from passages 25 to 45 in 12 well tissue culture plates (Costar) at 1.6 × 10^5^ cells/ml in DMEM, with all supplements. The culture medium was replaced every second day. Overnight grown cultures of AUS0004, ΔEFAU004_01209, the complemented strain and the triple mutant were diluted (1:100) and grown at 37 °C to an OD660 of 0.4. Bacteria were harvested by centrifugation and resuspended in DMEM to a concentration of 1 × 107 CFU/ml. For each strain, 1 ml bacterial suspension was added to the wells (100 bacteria per cell). Plates were centrifuged and incubated for 1 h at 37 °C. After incubation, monolayers were rinsed three times with DMEM/EMEM, and cells were lysed with 1% Triton X 100 (Merck, Darmstadt, Germany) in PBS for 5 min at room temperature. The adherent bacteria were quantified by plating serial dilutions on BHI agar plates and counting CFU. The inoculum was plated to determine viable counts. The assay was performed in triplicate and repeated twice

#### Ethics statement

The mouse experiments were performed under a protocol approved by the Institutional Animal Use and Care Committee at the Università Cattolica del Sacro Cuore, Rome, Italy (Protocol N° 903/2017 PR) and authorized by the Italian Ministry of Health, according to Legislative Decree 116/92, which implemented the European Directive 86/609/EEC on laboratory animal protection in Italy. Animal welfare was routinely checked by veterinarians of the Service for Animal Welfare. We confirm that all experiments were performed in accordance with relevant guidelines and regulations. Urine collection was approved and the need for informed consent was waived by the Institutional Review Board of Università Cattolica del Sacro Cuore, Policlinico Universitario Agostino Gemelli, I.R.C.C.S. (protocol number 49/18). All methods were carried out in accordance with relevant guidelines and regulations.

## Electronic supplementary material

Figures S1-S5, Tables S1-S5
