# Supplementary material for: Expression profiling in a mammalian host reveals the strong induction of genes encoding LysM domain-containing proteins in Enterococcus faecium
Source: Sci Rep. 2018 Aug 17;8:12412. doi: 10.1038/s41598-018-30882-z (PMC6098018; doi:10.1038/s41598-018-30882-z)
Supplement: Supplementary file 1 — Figures S1-S5, Tables S1-S5 [file 41598_2018_30882_MOESM1_ESM.docx]

**Expression profiling in a mammalian host reveals the strong induction of genes encoding LysM domain-containing proteins in *Enterococcus faecium***

**Margherita-Cacaci^1,2^*^#^*, Caroline-Giraud^1^*^#^*, Loïc-Leger^1^, Riccardo Torelli^2^, Cecilia Martini^2^, Brunella Posteraro^3^, Valentina Palmieri^4^, Maurizio Sanguinetti^2^*, Francesca Bugli^2≠^, and Axel Hartke^1≠^***

1Normandie Univ, UNICAEN, U2RM-Stress and Virulence, 14000 Caen, France

2 Institute of Microbiology, Università Cattolica del Sacro Cuore, Fondazione Policlinico Universitario-Agostino Gemelli, 00168, Rome, Italy.

3Institute of Public Health (Section of Hygiene), Università Cattolica del Sacro Cuore, Fondazione-Policlinico Universitario Agostino Gemelli, 00168, Rome, Italy.

4Physics Institute, Università Cattolica del Sacro Cuore, Fondazione Policlinico Universitario-Agostino Gemelli, 00168, Rome, Italy.

*Corresponding authors: [**axel.hartke@unicaen.fr**](mailto:axel.hartke@unicaen.fr) **; Maurizio.Sanguinetti@unicatt.it**

# These authors contributed equally to this work.

≠ These authors also contributed equally to this work.

**Figure S1.** Expression of the EFAU004_01209 gene in the Enterococcus faecium AUS0004 grown in brain heart infusion broth (BHI) (black bars) and mice peritoneum (24 h p.i) (grey bar) as assessed by real-time reverse transcription polymerase chain reaction. The level of the EFAU004_01209 transcript was tested in triplicate and normalized using the gyrA transcript level. Asterisks indicate points of statistical significance; *P < 05, Mann–Whitney test.

**
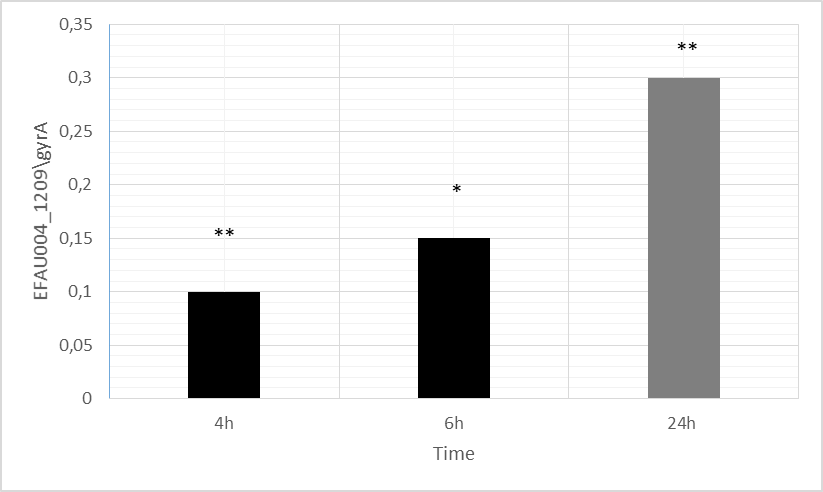
**

**Figure S2.** Number of colony forming unit (C.F.U.) of the bacteria inoculated in mice peritoneum

(grey bar) and of bacteria recovered after 24 hours infection (black bar). Serial dilution were plated on BHI agar to check viable bacteria.

**Figure S3.** Growth curves of *E. faecium* strains. Cells were grown in tubes in BHI without agitation at 37°C and growth was monitored at OD600. Filled circles: AUS0004; open triangles: triple mutant ΔEFAU004_0159-1150-494 ; filled squares : ΔEFAU004_01209. For clarity, only growth of the triple mutant is shown since growth of the single mutants ΔEFAU004_00494, ΔEFAU004_01059 and ΔEFAU004_01150 is similar to that of the triple mutant. Error bars denote standard deviation

.

**Figure S4**. **Sedimentation of bacterial cells**. E. faecium cultures were incubated at 37°C for 24 hours without shaking. The OD600 were comparable between the cultures after vortexing.


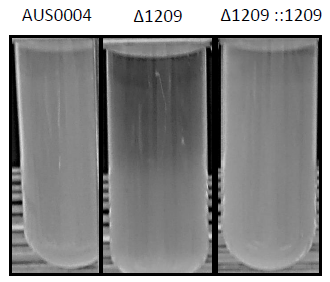


**Figure S5.** Western Blot with Anti-1209 polyclonal serum on whole cell lysates. M: Marker proteins; Lane 1: Aus0004; Lane 2: $\Delta EFAU004\_1209$ mutant strain negative control. Lane 3: ΔEFAU004_1059-1150-494 positive control.


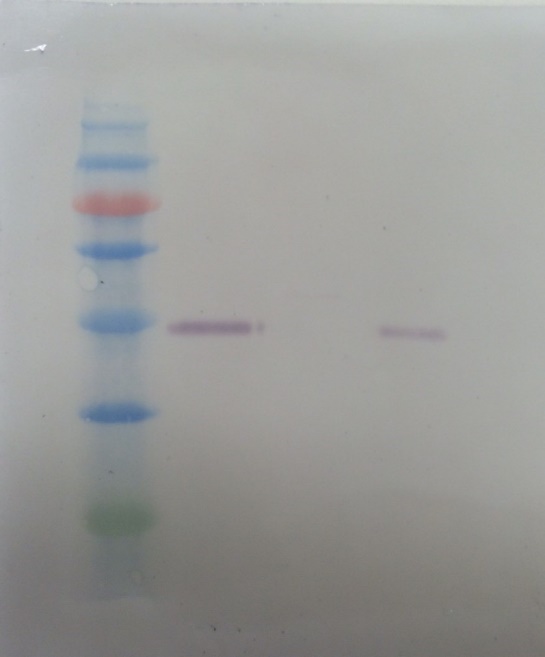


**45 kDa**

**M 1 2 3**

**Table S1. Strains used in this study**.

| **Strain** | **Description** | **Reference** |
| --- | --- | --- |
| AUS0004 | *E. faecium* wild-type strain- bloodstream infection | [14] |
| ΔEFAU004_00494 | AUS0004 strain with full deletion of EFAU004_00494 gene | this work |
| ΔEFAUOO4_1059 | AUS0004 strain with full deletion of EFAU004_01059 gene | this work |
| ΔEFAUOO4_1150 | AUS0004 strain with full deletion of EFAU004_01150 gene | this work |
| ΔEFAUOO4_1209 | AUS0004 strain with full deletion of EFAU004_01209 gene | this work |
| ΔEFAUOO4_1059-1150 double mutant | AUS0004 strain with full deletion of EFAU004_01059, EFAU004_01150 genes | this work |
| ΔEFAUOO4_1059-1150-494 triple mutant | AUS0004 strain with full deletion of EFAU004_01059, EFAU004_01150, EFAU004_00494 genes | this work |
| Δ1209 :1209 | AUS0004 strain with full deletion of EFAU004_01209 gene, complemented in *trans* on the chromosome | this work |
| *E. coli* Mach1-T1 | PetSumo vector host | Thermo Fisher Scientific |
| *E. coli* BL21(DE3) | Expression of recombinant His-Tag protein | Thermo Fisher Scientific |
| *E. coli* EC1000 | PwS3 vector host | [35] |
| JH2-2 | *E*.*faecalis* wild-type strain | [29] |

**Table S2.** **Plasmids used in this study.**

| **Plasmid** | **Description** | **Reference** |
| --- | --- | --- |
| PWS3 | Creation of deletion mutant in *E. faecium* AUS0004 | [14] |
| PetSumo | Expression of recombinant His-Tag proteins | Thermo Fisher Scientific |

**Table S3.** **Primers used in this study**.

| **PRIMER NAME** | **SEQUENCE 5'-3'** | **BP** | **TARGET** | **PURPOSE** |
| --- | --- | --- | --- | --- |
| ly494  fw1 | GGGGGATCGATTACCATGATATACTCAGAG | 30 | upstream EFAU004_00494 | deletion LysM 00494 in *E. faecium* AUS0004 |
| ly494  fw2 | CGCGAAGCTTCGTCTAGCTCCGCAAGTAGATACTGG | 36 | upstream EFAU004_00494 | deletion LysM 00494 in *E. faecium* AUS0004 |
| ly494  rv3 | TATCGGCTTACATATTAAACTCCTTTTTTTCTCCG | 35 | upstream EFAU004_00494 | deletion LysM 00494 in *E. faecium* AUS0004 |
| ly494  fw4 | GTTTAATATG**TAA**GCCGATAAAAGGAAAATGATGAAACCG | 40 | downstream EFAU004_00494 | deletion LysM 00494 in *E. faecium* AUS0004 |
| ly494  rv5 | CGCGGCGGCCGCGTACATGTCACGCCCACATTCTGAC | 35 | downstream EFAU004_00494 | deletion LysM 00494 in *E. faecium* AUS0004 |
| ly494  rv6 | CCGCCCATTTCCGTTTCGTATATCTCC | 27 | downstream EFAU004_00494 | deletion LysM 00494 in *E. faecium* AUS0004 |
| 1059  fw1 | CTCCTCGAACTAATCAGTTTTCTAAAGGCTG | 31 | upstream EFAU004_01059 | deletion LysM 01059 in *E. faecium* AUS0004 |
| 1059  fw2 | CGCGCTCGAGGATGCTGCGGCTCTTGCATAATCATAGC | 38 | upstream EFAU004_01059 | deletion LysM 01059 in *E. faecium* AUS0004 |
| 1059  fw4 | ATTTTTCATG**TAA**GATTCATTATTTGAACGTTTAAACACA | 40 | downstream EFAU004_01059 | deletion LysM 01059 in *E. faecium* AUS |
| 01150  fw1 | GAAGAAACTCTGTTTTATCTTTTTGACTTTTTGTC | 35 | upstream EFAU004_01150 | deletion LysM 01150 in *E. faecium* AUS0004 |
| 01550  fw2 | CGCGGAATTCGCAGCAGTATAGATTACTGTTGCTTCTTTA | 40 | upstream EFAU004_01150 | deletion LysM 01150 in *E. faecium* AUS0004 |
| 01150  rv3 | ATGAATCTTACATGAAAAATCTCCTTTATTCGTTAAAAAG | 40 | upstream EFAU004_01150 | deletion LysM 01150 in *E. faecium* AUS0004 |
| 01150  fw4 | ATTTTTCATG**TAA**GATTCATTTATTAATCAAACAGTTTAC | 40 | downstream EFAU004_01150 | deletion LysM 01150 in *E. faecium* AUS0004 |
| 01550  rv5 | CGGCGGCCGCGATTATCTGGATATCTTTGGGTTAAACGGC | 40 | downstream EFAU004_01150 | deletion LysM 01150 in *E. faecium* AUS0004 |
| 01550  rv6 | CCTTGGCTGAGCGTCTATCAAAACGTTACG | 30 | downstream EFAU004_01150 | deletion LysM 01150 in *E. faecium* AUS0004 |
| 1209  fw1 | GCCCTCTTGGAAATCTCTTTCGATATCTTC | 30 | upstream EFAU004_01209 | deletion LysM 01209 in *E. faecium* AUS0004 |
| 1209  fw2 | CGCGACTAGTGCCGATCGTACTGATAACGACTAAATCTAA | 40 | upstream EFAU004_01209 | deletion LysM 01209 in *E. faecium* AUS0004 |
| 1209rv3 | TAAAACAGTGTAATAATAGCCGCGCTTTGCGGCTTTTT | 38 | upstream EFAU004_01209 | deletion LysM 01209 in *E. faecium* AUS0004 |
| 1209  fw4 | GCTATTATTA**CAC**TGTTTTACCTCCTAAACTCTATCGTG | 39 | downstream EFAU004_01209 | deletion LysM 01209 in *E. faecium* AUS0004 |
| 01209rv5 | CGGCGGCCGCTTCGCAATCTGAAAGAAGATACGTCCTCCG | 40 | downstream EFAU004_01209 | deletion LysM 01209 in *E. faecium* AUS0004 |
| 1209rv6 | GGTCATCATCGTGTCACCGCTGCTTTCTC | 29 | downstream EFAU004_01209 | deletion LysM 01209 in *E. faecium* AUS0004 |
| 1209A | TATTTAATTCTTAATTCTGACCAGGGT | 28 | EFAU004_012090 forward | expression of recombinant EFAU004_01209 |
| 1209B | GTGAGCAGAAAGATAGACATCAATCTAC | 29 | EFAU004_012090 reverse | expression of recombinant EFAU004_01209 |
| A1150 | GCAATGTCTGTTCTTTTTGTG | 21 | EFAU004_01150 upstream | sequencing control of deletion |
| B 1150 | TTCACGTGTTAAAGTATTCCTCGTTGAAC | 29 | EFAU004_01150 upstream | sequencing control of deletion |
| C 1150 | GTAGTCATCGGTACATTCGTCC | 22 | EFAU004_01150 downstream | sequencing control of deletion |
| A1059 | TGTCCAGGGATGACATTGGCAATC | 22 | EFAU004_01059 upstream | sequencing control of deletion |
| B 1059 | GAGCGAACATCATTCTGTTAC | 21 | EFAU004_01059 upstream | sequencing control of deletion |
| C 1059 | CATCGAGCGAACATCATTCTGTTAC | 20 | EFAU004_01059 downstream | sequencing control of deletion |
| OCG118 | GCCGCTTGACGGATGTATTTCAATCC | 26 | EFAU004-RS0793  Upstream | Complementation EFAU004_01209 |
| OCG119 | CGCGggtaccCTAAACCTTGGAAAGCATATGGAG | 34 | EFAU004-RS0793  Upstream | Complementation EFAU004_01209 |
| OCG120 | GTACCGTTGCGCTTTATTCTATATGTCATGAACAAGAG | 38 | EFAU004-RS0793  Downstream | Complementation EFAU004_01209 |
| OCG121 | AGAATAAAGCGCAACGGTACTTTTCCCAGA | 30 | EFAU004_01209 forward | Complementation EFAU004_01209 |
| OCG122 | AAGACAAAGCTCAAGAAATCGGTCGAGCAGG | 31 | EFAU004_01209 reverse | Complementation EFAU004_01209 |
| OCG123 | CGATTTCTTGAGCTTTGTCTTGTAAACTCCGTTT | 34 | EFAU004-RS15385  upstream | Complementation EFAU004_01209 |
| OCG124 | CGCGCCGCGGGCCATGCAGGAGGTAAGATATTA | 33 | EFAU004-RS15385  Upstream | Complementation EFAU004_01209 |
| OCG125 | TTTTGCAGATGGACAAGAAAAACCC | 25 | EFAU004-RS15385  downstream | Complementation EFAU004_01209 |
| 1209 fw | CCGGCTGCAGATGAAGA | 15 | FW EFAU004_01209 | Real-time PCR |
| 1209 rv | GCAGAGACAACACCGACTTCC | 20 | RV EFAU004_01209 | Real-time PCR |
| fw gyrA | CGCCGATTGACCGGTTTGG | 20 | FW gyrA | Real-time PCR |
| Rv gyrA | CCAGACTTAAGACCTCACCG | 20 | RV gyrA | Real-time PCR |

**Table S4: Common *in vivo* induced genes.log FC2: average log2 threshold of the over-expressed genes found in both the *in vivo* versus BHI exponential phase and *in vivo* versus BHI stationary phase analyses; pvalue: average of pvalue between the two analyses.** The differentially expressed genes p-values are corrected for multiple testing error with a 5%false discovery rate. The correction used is Benjamini-Hochberg.

| **gene locus** | **description** | **logFC2** | **Pvalue** |
| --- | --- | --- | --- |
| EFAU004_00021 | ISL3 family transposase | 2,98 | 2,29E-08 |
| EFAU004_00022 | DegV family protein | 2,3 | 6,59E-05 |
| EFAU004_00026 | zinc transporter ZupT | 1,31 | 1,06E-02 |
| EFAU004_00073 | GMP synthase | 2,35 | 2,94E-04 |
| EFAU004_00086 | purine nucleoside phosphorylase | 1,69 | 5,57E-03 |
| EFAU004_00127 | Bacterial membrane protein YfhO | 2,02 | 1,69E-04 |
| EFAU004_00140 | chromosome partition protein SMC | 2,25 | 3,60E-06 |
| EFAU004_00141 | HAD superfamily hydrolase | 1,97 | 5,72E-05 |
| EFAU004_00142 | signal recognition particle-docking protein FtsY | 2,94 | 7,31E-08 |
| EFAU004_00148 | iron ABC transporter substrate-binding protein | 2,93 | 4,06E-05 |
| EFAU004_00149 | hypothetical protein | 4,67 | 2,67E-09 |
| EFAU004_00150 | hypothetical protein | 4,19 | 9,33E-08 |
| EFAU004_00168 | hypothetical protein | 1,49 | 5,05E-03 |
| EFAU004_00169 | BioY family protein | 2,48 | 3,28E-05 |
| EFAU004_00174 | calcineurin-like phosphoesterase | 1,54 | 4,90E-03 |
| EFAU004_00198 | peptide deformylase | 2,29 | 1,59E-05 |
| EFAU004_00200 | Polyribonucleotide nucleotidyltransferase | 1,76 | 5,96E-03 |
| EFAU004_00204 | MATE efflux family protein | 3,09 | 5,96E-03 |
| EFAU004_00218 | DEAD/DEAH box helicase | 2,4 | 5,14E-04 |
| EFAU004_00257 | isopentenyl-diphosphate delta-isomerase | 1,65 | 1,35E-03 |
| EFAU004_00261 | deoxynucleoside kinase | 2 | 3,99E-05 |
| EFAU004_00288 | peptide methionine sulfoxide reductase | 1,17 | 1,29E-02 |
| EFAU004_00313 | transporter, dicarboxylate/amino acid:cation symporter family protein | 1,79 | 9,95E-07 |
| EFAU004_00314 | hypothetical protein | 1,61 | 1,79E-03 |
| EFAU004_00334 | hypothetical protein | 2,11 | 9,02E-03 |
| EFAU004_00344 | LysR family transcriptional regulator | 2,84 | 1,49E-08 |
| EFAUà004_00391 | hypothetical protein | 2,5 | 1,95E-07 |
| EFAU004_00399 | TetR family transcriptional regulator | 2,11 | 1,11E-02 |
| EFAU004_00416 | MerR family transcriptional regulator | 1,81 | 1,31E-02 |
| EFAU004_00426 | sensor histidine kinase | 1,98 | 3,38E-03 |
| EFAU004_00434 | hypothetical protein | 4,75 | 1,14E-16 |
| EFAU004_00435 | ABC transporter ATP-binding protein | 4,08 | 3,24E-15 |
| EFAU004_00436 | peptide ABC transporter permease/transmembrane protein | 4,29 | 5,98E-14 |
| EFAU004_00437 | aldose 1-epimerase | 3,42 | 7,33E-09 |
| EFAU004_00438 | aldose 1-epimerase | 3,31 | 4,06E-10 |
| EFAU004_00439 | N-acetylmuramoyl-L-alanine amidase | 2,11 | 9,68E-06 |
| EFAU004_00449 | periplasmic solute binding protein | 5,43 | 4,70E-20 |
| EFAU004_00457 | ATP-dependent DNA helicase PcrA | 2,16 | 6,11E-05 |
| EFAU004_00468 | dihydrodipicolinate synthase | 1,54 | 8,51E-03 |
| EFAU004_00489 | ABC transporter ATP-binding protein | 2,67 | 1,29E-02 |
| EFAU004_00494 | LysM domain-containing protein | 3,96 | 6,58E-10 |
| EFAU004_00503 | pyridine nucleotide-disulfide oxidoreductase | 1,51 | 5,29E-03 |
| EFAU004_00528 | tellurite resistance protein | 3,04 | 6,53E-10 |
| EFAU004_00529 | 5-bromo-4-chloroindolyl phosphate hydrolysis protein | 1,95 | 6,27E-07 |
| EFAU004_00530 | nudix hydrolase, YffH family | 1,73 | 7,51E-04 |
| EFAU004_00532 | 5'-methylthioadenosine/S-adenosylhomocysteine nucleosidase | 2,21 | 1,10E-02 |
| EFAU004_00537 | ribose-phosphate pyrophosphokinase | 1,14 | 1,38E-02 |
| EFAU004_00542 | tRNA-specific 2-thiouridylase MnmA | 1,75 | 3,73E-03 |
| EFAU004_00544 | aspartate aminotransferase | 3,46 | 5,61E-05 |
| EFAU004_00545 | major facilitator superfamily transporter | 3,17 | 1,84E-09 |
| EFAU004_00548 | DNA topoisomerase III | 1,58 | 1,25E-03 |
| EFAU004_00555 | ArsC family protein | 1,39 | 6,69E-03 |
| EFAU004_00559 | putative D-methionine-binding lipoprotein MetQ | 2 | 8,97E-04 |
| EFAU004_00562 | cysteine desulfurase | 1,34 | 8,97E-04 |
| EFAU004_00563 | NifU family SUF system FeS assembly protein | 1,59 | 6,35E-03 |
| EFAU004_00564 | FeS assembly protein SufB | 3,02 | 6,27E-08 |
| EFAU004_00585 | putative foldase protein PrsA | 1,51 | 1,22E-02 |
| EFAU004_00609 | hypothetical protein | 1,8 | 4,34E-04 |
| EFAU004_00614 | sulfatase | 3,57 | 3,54E-10 |
| EFAU004_00615 | transcriptional activator Rgg/GadR/MutR | 2,46 | 4,32E-03 |
| EFAU004_00623 | peptidase M20D | 2,75 | 1,05E-03 |
| EFAU004_00626 | amino acid permease | 7,58 | 1,32E-31 |
| EFAU004_00633 | N-acetylmuramic acid 6-phosphate etherase | 1,6 | 8,74E-04 |
| EFAU004_00634 | PTS system, sugar-specific IIBC component | 2,11 | 1,41E-05 |
| EFAU004_00643 | hypothetical protein | 2,06 | 1,93E-04 |
| EFAU004_00644 | nicotinamide mononucleotide transporter PnuC | 3,02 | 6,75E-06 |
| EFAU004_00646 | hypothetical protein | 4,32 | 3,00E-15 |
| EFAU004_00647 | hypothetical protein | 1,63 | 6,21E-04 |
| EFAU004_00688 | site-specific tyrosine recombinase XerC-family | 1,53 | 3,76E-03 |
| EFAU004_00691 | MerR family transcriptional regulator | 2,3 | 2,46E-05 |
| EFAU004_00706 | CarD family transcriptional regulator | 1,43 | 1,15E-02 |
| EFAU004_00717 | DNA replication initiation control protein YabA | 1,87 | 1,32E-04 |
| EFAU004_00720 | anaerobic ribonucleoside-triphosphate reductase activating protein | 2,28 | 1,14E-03 |
| EFAU004_00721 | anaerobic ribonucleoside triphosphate reductase | 1,76 | 4,95E-03 |
| EFAU004_00725 | D-alanine--poly(phosphoribitol) ligase subunit 1 | 4,18 | 5,83E-15 |
| EFAU004_00726 | membrane bound O-acyl transferase | 3,97 | 8,20E-15 |
| EFAU004_00727 | D-alanine--poly(phosphoribitol) ligase subunit 2 | 4,4 | 4,87E-16 |
| EFAU004_00728 | D-alanyl-lipoteichoic acid synthetase, DltD | 4,81 | 8,92E-18 |
| EFAU004_00770 | cell division protein FtsA | 1,55 | 1,04E-03 |
| EFAU004_00771 | cell division protein FtsZ | 2,04 | 9,14E-04 |
| EFAU004_00855 | hypothetical protein | 2,13 | 1,99E-04 |
| EFAU004_00858 | glutaminase | 1,91 | 1,15E-02 |
| EFAU004_00865 | FtsW/RodA/SpoVE family cell cycle protein | 2,47 | 1,61E-03 |
| EFAU004_00866 | cell envelope-related function transcriptional attenuator | 4,84 | 8,09E-11 |
| EFAU004_00870 | penicillin binding protein transpeptidase domain protein | 1,54 | 1,15E-02 |
| EFAU004_00882 | DNA-binding transcriptional repressor MarR | 1,65 | 4,49E-04 |
| EFAU004_00917 | hypothetical protein | 5,02 | 7,63E-20 |
| EFAU004_00918 | hypothetical protein | 4,27 | 7,37E-14 |
| EFAU004_00919 | phosphomethylpyrimidine kinase | 4,44 | 1,81E-15 |
| EFAU004_00923 | sensor histidine kinase | 2,09 | 2,12E-05 |
| EFAU004_00926 | efflux ABC transporter permease | 3,55 | 4,20E-03 |
| EFAU004_00927 | ABC transporter ATP-binding protein | 2,45 | 3,70E-03 |
| EFAU004_00932 | LPXTG-motif protein cell wall anchor domain protein | 1,3 | 1,37E-02 |
| EFAU004_00940 | alpha-L-rhamnosidase | 1,93 | 1,24E-02 |
| EFAU004_00950 | 3-demethylubiquinone-9 3-methyltransferase | 2,83 | 3,55E-06 |
| EFAU004_00951 | transcriptional regulator, PSR protein | 3,5 | 2,71E-10 |
| EFAU004_00976 | Formate--tetrahydrofolate ligase | 2,69 | 1,48E-04 |
| EFAU004_00990 | hypothetical protein | 2 | 5,24E-04 |
| EFAU004_00994 | cell division protein DivIVA | 1,16 | 1,27E-02 |
| EFAU004_00996 | recombination protein U | 3 | 2,19E-09 |
| EFAU004_00997 | penicillin-binding protein 1A | 3,23 | 3,90E-08 |
| EFAU004_00999 | endonuclease III | 2,19 | 1,00E-04 |
| EFAU004_01019 | Obg family GTPase CgtA | 2,18 | 6,43E-03 |
| EFAU004_01021 | x-Pro dipeptidyl-peptidase | 2,07 | 3,56E-04 |
| EFAU004_01024 | cyclophilin type peptidyl-prolyl cis-trans isomerase | 3,32 | 3,56E-04 |
| EFAU004_01045 | amino acid/peptide transporter | 3,97 | 2,07E-07 |
| EFAU004_01059 | peptidoglycan-binding protein LysM | 9,23 | 3,75E-39 |
| EFAU004_01074 | ComE operon protein 2 | 2,04 | 9,28E-04 |
| EFAU004_01104 | Phosphoribosylamine--glycine ligase | 6,33 | 1,27E-26 |
| EFAU004_01105 | bifunctional purine biosynthesis protein PurH | 5,91 | 1,03E-22 |
| EFAU004_01106 | Phosphoribosylglycinamide formyltransferase | 5,64 | 5,71E-21 |
| EFAU004_01107 | Phosphoribosylformylglycinamidine cyclo-ligase | 5,58 | 3,80E-18 |
| EFAU004_01108 | Amidophosphoribosyltransferase | 5,74 | 1,31E-19 |
| EFAU004_01109 | Phosphoribosylformylglycinamidine synthase II | 5,25 | 8,23E-19 |
| EFAU004_01110 | Phosphoribosylformylglycinamidine synthase I | 5,57 | 6,04E-15 |
| EFAU004_01111 | phosphoribosylformylglycinamidine synthase subunit PurS | 3,53 | 4,74E-04 |
| EFAU004_01112 | Phosphoribosylaminoimidazole-succinocarboxamide synthase | 4,65 | 2,56E-10 |
| EFAU004_01115 | Adenylosuccinate lyase | 2,7 | 4,21E-03 |
| EFAU004_01116 | phosphoribosylaminoimidazole carboxylase, ATPase subunit | 2,74 | 1,35E-04 |
| EFAU004_01117 | phosphoribosylaminoimidazole carboxylase catalytic subunit | 4,77 | 1,35E-14 |
| EFAU004_01118 | xanthine permease | 2,18 | 2,40E-04 |
| EFAU004_01119 | xanthine phosphoribosyltransferase | 1,85 | 4,12E-03 |
| EFAU004_01141 | aminotransferase AlaT | 2,02 | 2,26E-05 |
| EFAU004_01150 | LysM domain-containing protein | 8,36 | 4,54E-35 |
| EFAU004_01178 | site-specific tyrosine recombinase XerC-family | 1,34 | 5,93E-03 |
| EFAU004_01186 | signal peptidase I | 1,44 | 8,33E-03 |
| EFAU004_01188 | methionine sulfoxide reductase MsrA | 1,82 | 1,79E-04 |
| EFAU004_01189 | hypothetical protein | 1,59 | 2,07E-03 |
| EFAU004_01195 | ABC transporter ATP-binding protein | 2,08 | 2,55E-04 |
| EFAU004_01209 | LysM domain-containing protein | 2,02 | 1,20E-04 |
| EFAU004_01225 | hypothetical protein | 3,7 | 2,88E-11 |
| EFAU004_01226 | RNA polymerase sigma factor RpoD | 1,69 | 9,21E-04 |
| EFAU004_01228 | cation transporter E1-E2 family ATPase | 2,21 | 7,95E-04 |
| EFAU004_01229 | membrane protein | 2,54 | 3,72E-06 |
| EFAU004_01230 | hypothetical protein | 1,81 | 4,65E-03 |
| EFAU004_01231 | alpha/beta hydrolase | 2,63 | 4,51E-07 |
| EFAU004_01232 | ysyl-tRNA synthetase | 1,96 | 5,45E-03 |
| EFAU004_01233 | translation elongation factor Tu | 1,45 | 4,19E-03 |
| EFAU004_01239 | acetyl-CoA | 2,12 | 1,69E-04 |
| EFAU004_01240 | hydroxymethylglutaryl-CoA synthase | 3,66 | 6,93E-12 |
| EFAU004_01244 | phosphomethylpyrimidine kinase | 2,72 | 3,72E-08 |
| EFAU004_01247 | hypothetical protein | 2,25 | 7,71E-05 |
| EFAU004_01253 | glyoxalase family protein | 1,41 | 8,89E-03 |
| EFAU004_01267 | L-ribulose-5-phosphate 4-epimerase | 3,42 | 2,35E-05 |
| EFAU004_01269 | 3-keto-L-gulonate-6-phosphate decarboxylase | 3,13 | 4,43E-04 |
| EFAU004_01273 | L-ascorbate 6-phosphate lactonase, UlaG | 2,83 | 1,89E-04 |
| EFAU004_01289 | nucleic acid-binding protein | 2,04 | 8,91E-04 |
| EFAU004_01290 | LysR family transcriptional regulator | 3,3 | 3,48E-10 |
| EFAU004_01296 | xanthine/uracil/vitamin C permease | 4,91 | 1,64E-13 |
| EFAU004_01297 | HAD superfamily hydrolase | 2,99 | 1,14E-07 |
| EFAU004_01299 | Penicillin binding protein transpeptidase domain protein | 4,17 | 1,26E-14 |
| EFAU004_01325 | MarR family transcriptional regulator | 2,65 | 3,19E-04 |
| EFAU004_01326 | excinuclease ABC | 2,24 | 7,42E-04 |
| EFAU004_01337 | hemolysin | 2,22 | 6,35E-06 |
| EFAU004_01343 | 23S rRNA (uracil-5-)-methyltransferase | 2,21 | 9,46E-03 |
| EFAU004_01346 | transcriptional regulator | 2,1 | 1,61E-05 |
| EFAU004_01355 | LuxR family DNA-binding response regulator | 1,8 | 2,81E-03 |
| EFAU004_01357 | membrane protein | 1,31 | 8,81E-03 |
| EFAU004_01359 | putative aminodeoxychorismate lyase | 1,84 | 2,00E-04 |
| EFAU004_01362 | ISL3 family transposase | 4,35 | 1,10E-04 |
| EFAU004_01492 | hypothetical protein | 2,08 | 4,45E-05 |
| EFAU004_01505 | phosphosugar isomerase transcriptional regulator | 2,37 | 6,08E-04 |
| EFAU004_01586 | LacI family transcriptional regulator | 2,08 | 6,50E-07 |
| EFAU004_01587 | esterase | 1,68 | 7,37E-04 |
| EFAU004_01589 | IS66 family transposase | 1,67 | 1,11E-03 |
| EFAU004_01601 | N-acetylmuramoyl-L-alanine amidase | 1,94 | 8,94E-04 |
| EFAU004_01603 | Glycosyl hydrolases family 18 | 2,15 | 1,06E-04 |
| EFAU004_01605 | Prophage endopeptidase tail | 1,66 | 1,04E-03 |
| EFAU004_01606 | Phage tail protein | 2,43 | 3,53E-04 |
| EFAU004_01607 | Phage-related minor tail protein | 1,63 | 1,11E-03 |
| EFAU004_01608 | hypothetical protein | 2,52 | 3,38E-04 |
| EFAU004_01609 | phage major tail protein | 1,99 | 6,41E-04 |
| EFAU004_01610 | hypothetical protein | 1,97 | 5,77E-03 |
| EFAU004_01611 | phage protein, HK97 gp10 family | 2,04 | 7,71E-03 |
| EFAU004_01613 | phage protein | 2,56 | 6,99E-07 |
| EFAU004_01614 | major capsid protein | 2,32 | 1,03E-05 |
| EFAU004_01615 | peptidase S14 | 2,33 | 2,37E-05 |
| EFAU004_01616 | Phage portal protein | 1,79 | 4,71E-04 |
| EFAU004_01617 | phage terminase-like protein large subunit | 1,39 | 4,71E-04 |
| EFAU004_01628 | hypothetical protein | 3,38 | 1,79E-05 |
| EFAU004_01630 | phage-associated helicase | 2,8 | 4,16E-05 |
| EFAU004_01632 | phage DNA polymerase | 2,64 | 2,25E-05 |
| EFAU004_01638 | hypothetical protein | 1,94 | 1,93E-03 |
| EFAU004_01639 | hypothetical protein | 1,63 | 2,25E-05 |
| EFAU004_01640 | DNA polymerase I | 3,14 | 4,45E-06 |
| EFAU004_01642 | hypothetical protein | 2,1 | 1,72E-05 |
| EFAU004_01643 | hypothetical protein | 2,29 | 5,22E-06 |
| EFAU004_01644 | Glyoxalase-like domain protein | 1,34 | 1,10E-02 |
| EFAU004_01645 | hypothetical protein | 1,54 | 4,72E-03 |
| EFAU004_01646 | hypothetical protein | 2,09 | 3,65E-04 |
| EFAU004_01647 | Eco29kI restriction endonuclease | 2,48 | 1,95E-05 |
| EFAU004_01648 | Site-specific DNA methylase | 2,23 | 1,31E-04 |
| EFAU004_01672 | phosphoglucosamine mutase | 1,7 | 2,12E-03 |
| EFAU004_01704 | L,D-carboxypeptidase A | 1,45 | 1,85E-04 |
| EFAU004_01729 | arginine repressor protein | 2,59 | 4,41E-07 |
| EFAU004_01751 | peptidase, S54 family protein | 1,23 | 1,63E-02 |
| EFAU004_01753 | ferrichrome transport system permease FhuG | 1,36 | 6,37E-03 |
| EFAU004_01754 | ferrichrome transport system permease FhuB | 1,59 | 1,30E-02 |
| EFAU004_01756 | periplasmic binding protein | 4,03 | 3,65E-14 |
| EFAU004_01773 | hypothetical protein | 2,33 | 6,98E-05 |
| EFAU004_01774 | glycerophosphodiester phosphodiesterase family protein | 2,88 | 3,39E-07 |
| EFAU004_01779 | ISEfm1, transposase | 5,76 | 7,30E-08 |
| EFAU004_01789 | sulfatase | 2 | 1,98E-04 |
| EFAU004_01790 | hypothetical protein | 1,4 | 7,86E-04 |
| EFAU004_01791 | hypothetical protein | 1,86 | 3,24E-04 |
| EFAU004_01838 | glycoside hydrolase, family 32 | 1,75 | 3,09E-03 |
| EFAU004_01867 | cell wall surface anchor family protein | 2,89 | 8,94E-04 |
| EFAU004_01870 | tRNA (guanine-N1)-methyltransferase | 1,22 | 8,35E-03 |
| EFAU004_01878 | signal recognition particle protein | 2,22 | 5,17E-03 |
| EFAU004_01882 | sensory box histidine kinase | 1,67 | 6,66E-03 |
| EFAU004_01883 | cation transporter E1-E2 family ATPase | 4,1 | 2,86E-15 |
| EFAU004_01886 | phosphate-binding protein | 3,78 | 2,87E-06 |
| EFAU004_01891 | ISL3 family transposase | 1,48 | 2,85E-03 |
| EFAU004_01906 | hypothetical protein | 2,48 | 6,94E-07 |
| EFAU004_01921 | cadmium-translocating P-type ATPase | 4,75 | 3,28E-13 |
| EFAU004_01925 | asparaginyl-tRNA synthetase | 2,13 | 7,12E-03 |
| EFAU004_01926 | aspartate aminotransferase | 1,71 | 2,82E+00 |
| EFAU004_01927 | hypothetical protein | 1,5 | 2,25E-03 |
| EFAU004_01928 | DnaQ family exonuclease/DinG family helicase | 1,69 | 7,97E-04 |
| EFAU004_01933 | hypothetical protein | 2,82 | 1,04E-03 |
| EFAU004_01934 | D-alanyl-D-alanine carboxypeptidase | 3,52 | 4,15E-11 |
| EFAU004_01937 | fibronectin-binding protein | 2,98 | 8,29E-11 |
| EFAU004_01938 | HD domain-containing protein | 3,7 | 4,79E-07 |
| EFAU004_01941 | putative OxaA-like protein | 2,78 | 5,04E-04 |
| EFAU004_02004 | 6-phospho-beta-glucosidase | 2,1 | 3,81E-05 |
| EFAU004_02010 | ABC transporter ATP-binding protein | 1,84 | 4,65E-04 |
| EFAU004_02016 | glycosyl transferase family protein | 2,59 | 3,00E-07 |
| EFAU004_02018 | Xaa-Pro dipeptidase | 3,06 | 3,91E-05 |
| EFAU004_02025 | hypothetical protein | 3,24 | 1,10E-08 |
| EFAU004_02026 | PspC domain-containing protein | 2,41 | 2,36E-09 |
| EFAU004_02027 | hypothetical protein | 3,31 | 2,50E-07 |
| EFAU004_02037 | efflux ABC transporter permease | 2,78 | 4,82E-05 |
| EFAU004_02040 | Preprotein translocase subunit SecA | 1,88 | 2,28E-03 |
| EFAU004_02046 | hypothetical protein | 1,33 | 5,52E-03 |
| EFAU004_02077 | SorC family transcriptional regulator | 3,14 | 8,38E-13 |
| EFAU004_02082 | DNA-directed RNA polymerase subunit delta | 1,58 | 3,80E-03 |
| EFAU004_02092 | V-type sodium ATP synthase subunit J | 2,12 | 1,44E-05 |
| EFAU004_02093 | V-type ATP synthase subunit D | 1,57 | 8,63E-04 |
| EFAU004_02094 | V-type ATP synthase subunit B | 1,73 | 7,31E-04 |
| EFAU004_02108 | recombination and DNA strand exchange inhibitor protein | 1,55 | 3,13E-03 |
| EFAU004_02155 | hypothetical protein | 1,6 | 1,01E-02 |
| EFAU004_02157 | hypothetical protein | 1,8 | 1,52E+00 |
| EFAU004_02193 | Carbonic anhydrase/acetyltransferase | 1,68 | 5,69E-04 |
| EFAU004_02198 | Ribonuclease J 1 | 2,15 | 5,99E-06 |
| EFAU004_02232 | cytidine/deoxycytidylate deaminase family protein | 1,81 | 8,38E-04 |
| EFAU004_02235 | rRNA (cytosine-C(5)-)-methyltransferase | 2,28 | 3,26E-05 |
| EFAU004_02244 | Ribonuclease J 2 | 1,5 | 6,31E-03 |
| EFAU004_02251 | hypothetical protein | 1,21 | 1,95E+00 |
| EFAU004_02253 | glycerol-3-phosphate dehydrogenase | 1,84 | 2,37E-04 |
| EFAU004_02272 | hypothetical protein | 2,51 | 3,87E-03 |
| EFAU004_02276 | IS200/IS605 family transposase | 5,42 | 1,48E-21 |
| EFAU004_02277 | ABC transporter ATP-binding protein | 6,42 | 7,42E-26 |
| EFAU004_02278 | ABC transporter ATP-binding protein | 7,24 | 1,24E-28 |
| EFAU004_02279 | oligopeptide ABC transporter, permease protein OppC family protein | 6,72 | 4,73E-27 |
| EFAU004_02280 | oligopeptide ABC superfamily ATP binding cassette transporter | 6,45 | 4,98E-22 |
| EFAU004_02281 | hypothetical protein | 2,43 | 1,47E-05 |
| EFAU004_02282 | oligopeptide ABC superfamily ATP binding cassette transporter | 7,07 | 2,39E-27 |
| EFAU004_02284 | glycerol dehydrogenase | 6,9 | 3,13E-30 |
| EFAU004_02285 | ribosomal RNA large subunit methyltransferase N | 3,62 | 5,87E-07 |
| EFAU004_02286 | cell division protein FtsK | 2,29 | 5,46E-05 |
| EFAU004_02292 | Collagen-binding protein | 2,56 | 4,88E-03 |
| EFAU004_02306 | acetyltransferase | 2,49 | 2,54E-03 |
| EFAU004_02308 | signal peptidase I | 3,38 | 8,76E-11 |
| EFAU004_02309 | hypothetical protein | 2,28 | 2,94E-03 |
| EFAU004_02310 | hypothetical protein | 2,9 | 6,56E-04 |
| EFAU004_02314 | CobB/CobQ-like glutamine amidotransferase domain protein | 1,26 | 6,91E-03 |
| EFAU004_02433 | Substrate binding domain of ABC-type glycine betaine transport system | 3,57 | 3,04E-08 |
| EFAU004_02434 | glycine betaine transporter ATP-binding subunit | 4,71 | 3,76E+00 |
| EFAU004_02454 | L-serine dehydratase, iron-sulfur-dependent subunit alpha | 2,13 | 1,93E-08 |
| EFAU004_02456 | hypothetical protein | 1,65 | 8,73E-05 |
| EFAU004_02486 | HAD superfamily hydrolase | 2,37 | 1,52E-04 |
| EFAU004_02490 | acyltransferase | 2,44 | 1,10E-05 |
| EFAU004_02491 | methyltransferase small domain-containing protein | 1,83 | 2,90E-04 |
| EFAU004_02493 | acyltransferase | 3,31 | 1,83E-06 |
| EFAU004_02509 | ABC transporter ATP-binding protein | 3,45 | 2,46E-11 |
| EFAU004_02519 | adenine deaminase | 3,52 | 2,36E-08 |
| EFAU004_02548 | glucuronate isomerase | 1,23 | 1,04E-02 |
| EFAU004_02552 | hypothetical protein | 3,69 | 1,27E-12 |
| EFAU004_02553 | penicillin-binding protein | 4,66 | 2,59E-17 |
| EFAU004_02554 | 23S rRNA pseudouridine synthase D | 1,61 | 3,80E-03 |
| EFAU004_02590 | TetR family transcriptional regulator | 1,41 | 4,82E-03 |
| EFAU004_02598 | chaperonin HslO | 1,22 | 1,27E-02 |
| EFAU004_02599 | ATP-dependent metalloprotease | 1,42 | 7,13E-03 |
| EFAU004_02600 | Hypoxanthine-guanine phosphoribosyltransferase | 1,43 | 3,99E-03 |
| EFAU004_02609 | ErfK/YbiS/YcfS/YnhG family protein | 2,53 | 1,22E-06 |
| EFAU004_02613 | NlpC/P60 family lipoprotein | 5 | 9,13E-19 |
| EFAU004_02618 | serine/threonine-protein kinase | 2,65 | 5,88E-05 |
| EFAU004_02619 | serine/threonine phosphatase | 1,71 | 9,70E-03 |
| EFAU004_02620 | Ribosomal RNA small subunit methyltransferase B | 1,87 | 3,32E-03 |
| EFAU004_02621 | Methionyl-tRNA formyltransferase | 1,75 | 9,11E-03 |
| EFAU004_02622 | Peptide deformylase | 1,55 | 7,95E-03 |
| EFAU004_02623 | Primosomal protein N' | 1,52 | 2,22E-03 |
| EFAU004_02624 | DNA-directed RNA polymerase subunit omega | 1,39 | 4,60E-03 |
| EFAU004_02635 | ABC transporter permease | 5,52 | 8,75E-17 |
| EFAU004_02636 | ABC transporter ATP-binding protein | 5,68 | 2,77E-20 |
| EFAU004_02637 | periplasmic solute binding family protein | 5,31 | 7,15E-20 |
| EFAU004_02641 | 2',3'-cyclic-nucleotide 2'-phosphodiesterase | 1,58 | 1,50E-03 |
| EFAU004_02645 | hypothetical protein | 3,21 | 3,49E-12 |
| EFAU004_02649 | GNAT family acetyltransferase | 1,92 | 6,19E-04 |
| EFAU004_02663 | gamma-glutamyl phosphate reductase | 1,61 | 1,54E-03 |
| EFAU004_02665 | hypothetical protein | 1,25 | 8,79E-03 |
| EFAU004_02691 | ABC transporter ATP-binding protein | 1,69 | 3,89E-03 |
| EFAU004_02694 | sensor histidine kinase | 1,82 | 3,69E-04 |
| EFAU004_02695 | response regulator receiver domain-containing protein | 1,95 | 3,36E-04 |
| EFAU004_02696 | ABC-2 family transporter protein | 1,99 | 1,91E-04 |
| EFAU004_02697 | bacitracin transport ATP-binding protein BcrA | 1,67 | 3,69E-04 |
| EFAU004_02759 | CobW/P47K family protein | 1,34 | 1,03E-02 |
| EFAU004_02762 | periplasmic solute binding family protein | 1,3 | 1,90E-03 |
| EFAU004_02768 | 30S ribosomal protein S14 | 1,75 | 4,77E-03 |
| EFAU004_02774 | D-alanyl-D-alanine dipeptidase | 4,16 | 2,25E-17 |
| EFAU004_02775 | D-alanine--D-lactate ligase | 4,28 | 3,86E-12 |
| EFAU004_02776 | VanH protein, D-specific alpha-keto acid dehydrogenase | 3,75 | 2,46E-08 |
| EFAU004_02777 | vancomycin B-type resistance protein VanW | 4,74 | 2,95E-12 |
| EFAU004_02778 | VanY protein, D-alanyl-D-alanine carboxypeptidase | 4,51 | 7,42E-11 |
| EFAU004_02779 | sensor histidine kinase VanSB | 1,95 | 6,33E-05 |
| EFAU004_02786 | Helix-turn-helix domain protein | 2,65 | 3,82E-11 |
| EFAU004_02798 | TraG/TraD family protein | 2,17 | 4,33E-03 |
| EFAU004_02799 | hypothetical protein | 5,72 | 2,63E-04 |
| EFAU004_02800 | hypothetical protein | 6,58 | 5,00E-07 |
| EFAU004_02801 | hypothetical protein | 8,46 | 1,15E-11 |
| EFAU004_02802 | ABC transporter ATP-binding protein | 4,06 | 1,59E-13 |
| EFAU004_02803 | hypothetical protein | 4,65 | 8,03E-12 |
| EFAU004_02804 | ABC transporter ATP-binding protein | 3,48 | 2,01E-06 |
| EFAU004_02805 | Cobalt transport protein | 3,46 | 1,75E-03 |
| EFAU004_02807 | hypothetical protein | 2,98 | 1,70E-03 |
| EFAU004_02808 | iron dependent repressor, DNA binding domain protein | 3,75 | 3,33E-09 |
| EFAU004_02809 | adhesion lipoprotein | 5,06 | 1,24E-17 |
| EFAU004_02810 | metal ion ABC transporter permease | 3,1 | 1,83E-04 |
| EFAU004_02812 | ABC transporter ATP-binding protein | 3,08 | 1,83E-04 |
| EFAU004_02814 | 30S ribosomal protein S9 | 3,03 | 3,07E-05 |
| EFAU004_02838 | cell wall surface adhesion protein | 3,48 | 6,32E-11 |
| EFAU004_02855 | Inosine-5'-monophosphate dehydrogenase | 2,24 | 3,74E-03 |
| EFAU004_02859 | sporulation initiation inhibitor protein Soj | 1,75 | 2,33E-04 |
| EFAU004_02860 | Ribosomal RNA small subunit methyltransferase G | 1,64 | 3,58E-03 |
| EFAU004_02872 | 16S ribosomal RNA | 2,75 | 9,62E-01 |
| EFAU004_02887 | tRNA | 6,7 | 3,14E-20 |
| EFAU004_02902 | tRNA | 1,42 | 7,47E-03 |
| EFAU004_02912 | tRNA | 6,28 | 7,58E-14 |
| EFAU004_03017 | tRNA | 6,18 | 7,67E-21 |
| EFAU004_03037 | tRNA | 3,51 | 1,05E-09 |
| EFAU004_03042 | tRNA | 4,95 | 3,63E-06 |
| EFAU004_03047 | tRNA | 6,98 | 1,64E-20 |
| EFAU004_03052 | tRNA | 5,8 | 4,62E-15 |
| EFAU004_03057 | tRNA | 7 | 8,46E-22 |
| EFAU004_03077 | tRNA | 5,31 | 7,18E-10 |
| EFAU004_03087 | tRNA | 2,49 | 5,73E-03 |
| EFAU004_03172 | tRNA | 3,26 | 4,29E-09 |
| EFAU004_p1002 | regulatory protein TraE1 | 1,99 | 9,45E-03 |
| EFAU004_p1009 | sortase family protein | 2,48 | 3,93E-06 |
| EFAU004_p1010 | cell wall surface anchor family protein | 2,54 | 4,04E-06 |
| EFAU004_p1011 | sortase family protein | 2,24 | 2,80E-05 |
| EFAU004_p1012 | hypothetical protein | 1,69 | 1,16E-05 |
| EFAU004_p1013 | hypothetical protein | 2,65 | 2,02E-06 |
| EFAU004_p1018 | hypothetical protein | 2,41 | 1,15E-03 |
| EFAU004_p1019 | hypothetical protein | 1,86 | 1,34E-03 |
| EFAU004_p1025 | ATPase | 2,07 | 3,93E-05 |
| EFAU004_p1033 | hypothetical protein | 1,97 | 3,77E-03 |
| EFAU004_p1038 | DNA topoisomerase III | 2,99 | 1,72E-05 |
| EFAU004_p1042 | single-strand binding protein | 2,86 | 9,03E-09 |
| EFAU004_p1045 | hypothetical protein | 4,25 | 1,39E-08 |
| EFAU004_p1058 | ImpB/MucB/SamB family protein | 2,68 | 6,56E-04 |
| EFAU004_p1061 | replication control protein PrgN | 2,04 | 8,86E-03 |

**Table S5: Count per million reads (cpm) normalized according to the library seize.** The genes having a count above 1 cpm were kept for calculating the average cpm.

|  | experimental condition | | |
| --- | --- | --- | --- |
| gene locus | BHI exponential phase | BHI stationary phase | in vivo |
| EFAU004_00494 | 21,8289908 | 83,4935513 | 666,521862 |
| EFAU004_01059 | 5,38402678 | 24,0568395 | 6784,84111 |
| EFAU004_01150 | 6,04835011 | 20,3718175 | 3670,74163 |
| EFAU004_01209 | 151,885301 | 228,069084 | 750,545624 |
| average cpm for all genes | 415,84014 | 327,96218 | 411,122148 |
